# Algometer Assessment of Pressure Pain Threshold After Onabotulinumtoxin-A and Physical Therapy Treatments in Patients With Chronic Migraine: An Observational Study

**DOI:** 10.3389/fpain.2022.770397

**Published:** 2022-02-10

**Authors:** Manuela Deodato, Antonio Granato, Marta Ceschin, Alessandra Galmonte, Paolo Manganotti

**Affiliations:** ^1^Department of Medical, Surgical and Health Sciences, University of Trieste, Trieste, Italy; ^2^Department of Life Sciences, University of Trieste, Trieste, Italy; ^3^Azienda Sanitaria Universitaria Giuliano Isontina, Trieste, Italy

**Keywords:** chronic migraine, pressure pain threshold, onabotulinumtoxin-A, physical therapy, widespread pressure hyperalgesia, central sensitization, algometer, trigeminal area

## Abstract

The purpose of this study was to evaluate pain hypersensitivity in chronic migraine patients 3 months after undergoing onabotulinumtoxin-A therapy, physical therapy (PT), or the combination of the two. Pressure pain threshold (PPT) was assessed in accordance with Andersen's guidelines, focusing on five muscles in the trigeminocervical area (namely, trapezius, levator scapulae, temporalis, sub-occipitalis, and scalenus medius) and one muscle outside of the area, (i.e., tensor fasciae latae). Moreover, three headache parameters, namely, attack frequency, duration, and pain intensity, were recorded in an *ad hoc* diary kept by the patients. A total of 30 patients were included in three treatment groups: 1. onabotulinumtoxin-A therapy, 2. PT, and 3. a combination of onabotulinumtoxin-A and PT. The results show that, at the final assessment, the PPT was significantly reduced in the combined treatment group compared to the two single-therapy groups. As regards headache parameters, frequency and duration of the attacks were decreased significantly in all three treatment groups, whereas in pain intensity, the reduction was statistically significant in the combined treatment group and the onabotulinumtoxin-A therapy. Results suggest that a better pain modulation in patients with chronic migraine can be achieved with a combined treatment of onabotulinumtoxin-A and physical therapy. Indeed, the combination of both pharmacological and non-pharmacological treatments results in the reduction of both headache-related parameters and widespread pressure hyperalgesia.

## Introduction

Migraine is one of the most severe and burdensome types of headaches. This multifaceted and fragmented burden of migraine is often related to a complex physiopathology. Indeed, migraine is characterized by a lack of habituation that could lead to hyper-excitability of the brain and to central sensitization, which may result in hyperalgesia of the trigeminal system (1–3).

A key aspect of this imbalance of excitatory-inhibitory modulation leads to the expansion of the pain-affected area and an increase in frequency and intensity of migraine (4, 5). Clinically, alterations in descending pain inhibition manifest through widespread pressure hyperalgesia (2, 6, 7). Indeed, several studies identify lower pressure pain threshold (PPT) in the trigeminocervical complex and throughout the body in patients with chronic migraine with respect to that identified in the same muscles in healthy controls (8–11). These studies also show lower PPT in female patients with respect to male patients, while no difference was observed between episodic and chronic forms, between migraine and tension-type headache, or between symptomatic and non-symptomatic sides. Extensive research has been carried out on the PPT in chronic migraine, but no previous study has assessed PPT variation following different types of treatment. Moreover, although a conspicuous amount of literature has underlined the importance of a multidisciplinary approach, no earlier work has evaluated the PPT following combined treatments in this sense (8–10).

At present, the most common prophylactic approach to migraine consists of pharmacological and non-pharmacological desensitization treatments. As regards pharmacological treatments, onabotulinumtoxin-A represents one of the most frequent therapies for chronic migraine (12–15); physical therapy (PT) is usually considered an effective non-pharmacological alternative (16, 17).

This study investigated whether a combination of pharmacological and non-pharmacological therapy is able to achieve a higher desensitization rate in patients who suffered from chronic migraine than standard single-type therapy approaches. To that end, our study assessed each group of patients 3 months prior and 3 months following three types of treatments, i.e., (a) treatment with sole onabotulinumtoxin-A, (b) treatment with sole PT, and (c) treatment with a combination of onabotulinumtoxin-A and PT. Assessment parameters were as follows: (1) PPT in five muscles of the trigeminocervical complex and one muscle outside said areas and (2) headache parameters, such as total days of headache per month, duration of attacks, and intensity of pain.

## Method

An observational retrospective cohort study was chosen to evaluate the usual clinical practices regarding chronic migraine patients. The study was carried out by the Neurology Clinic of ASUGI (Regional Health Authority) and the Department of Physical Therapy of the University of Trieste, and it was approved by the institutional review board of Comitato etico unico regionale (CEUR, i.e., Regional Ethics Committee; project identification code 143_2018; ID 2432). The research was conducted according to the Code of Ethics of the World Medical Association (Declaration of Helsinki), and informed consent was signed by all the patients.

At T0, patients underwent an anamnestic and neurological examination by two neurology specialists. The following criteria of inclusion were applied: diagnosis of chronic migraine by criteria of ICDH3-beta (18); age over 18. Exclusion criteria were pregnancy; important psychiatric conditions; important pathologies, such as traumas, tumors, or infections; important surgical procedures over the previous 12 months; non-pharmacological or pharmacological prophylactic treatments over the previous 3 months; contraindications relating to onabotulinumtoxin-A and/or PT; cervical spine diseases; and the presence of ictal or interictal cutaneous allodynia (12-item Allodynia Symptom Checklist questionnaire). Prior to data collection, a diary was handed to each patient asking them to record the following information: attack frequency (headache days per month), duration, intensity, and the number of symptomatic medications taken each month. All patients were re-evaluated after 1 month (baseline period). In accordance with the diagnostic criteria of ICDH3-beta (18), the following eligibility criteria were applied: headache's frequency had to be ≥15 days per month and ≥50% of headache days had to be characterized by migraine crises (≥4 h of continuous severe headache, or 1 h of headache followed by intake of symptomatic medication).

Data concerning PPT were then collected by a physiotherapist before (T1) and after (T2) each treatment. Patients were also asked to continue recording headache parameters throughout the entire period of treatment. In accordance with the guidelines of the International Headache Society (19), each patient was allowed to take symptomatic medications in case of severe headache two times a week at most. In addition, patients had to register each medication's dosage and intake frequency in their diary.

### Pressure Pain Threshold

A Somedic algometer was selected for its reliability and validity to assess PPT (8, 10, 20–22). Somedic has a small surface that allows for easy and accurate evaluation performed on craniofacial muscles. The evaluation was carried out in accordance with Andersen's guidelines for the standardization of PPT assessment in craniofacial muscles (8).

Pressure pain threshold was assessed bilaterally in five muscles of the trigeminocervical complex (i.e., trapezius, levator scapulae, temporalis, sub-occipitalis, and scalenus medius) and one muscle outside said area (i.e., tensor fascia latae). Prior to the muscle evaluation, a trial measurement was performed on the wrist of each patient to familiarize them with the procedure (8). Subsequently, three consecutive measures were taken for each muscle with a 1-min interval between each measure, following the same order of measurement. The increasing rate was ~30 kPa/s. Patients were asked to press the stop button of the algometer when the perceived pressure changed into pain (8). PPT measurements were carried out in the following order: temporalis, scalenus medius, and tensor fasciae latae were examined with patients positioned on their left flank, using cushions to allow for muscle relaxation. Subsequently, patients were positioned on their right flank, with pillows to allow for muscle relaxation, and muscle measurements of temporalis, scalenus medius, and tensor fasciae latae on the left side were performed. Finally, patients moved into the prone position with pillows, and the following muscles were assessed: sub-occipitalis right, trapezius right, levator scapulae right, sub-occipitalis left, trapezius left, and levator scapulae left. During the measurement procedure, the physiotherapist would apply counter-pressure with one hand to immobilize the patient's head (8).

In line with previous studies, measurements were conducted exclusively during the pain-free periods (i.e., 3 days after the latest migraine attack) in all subjects (23–25), and, in the case of female patients, only in the late follicular phase (i.e., in the period between the day after the end of the menstruation and the day before the start of the ovulation) (26, 27). We allowed, only in very few cases, to carry out the assessment in the presence of mild intensity headache (numeric pain rating scale 1/2 out of 10) that does not require taking symptomatic medications.

The cohort was then included in one of the three treatment groups: (a) onabotulinumtoxin-A (BoNT-A) only, (b) PT only, and (c) onabotulinumtoxin-A and PT combined (BoNT-A+PT). The neurologist explained the different therapies, then the treatment was chosen on the basis of the patient's choice and of the specialist's opinion, as usual in the normal clinical practice. A total of 30 patients were enrolled, i.e., 10 patients per group (size of 0.5, alfa error 0.05, and power beta 0.8).

The physical therapy group (PT) consisted of 10 women (mean age 52.5 ± 17); the onabotulinumtoxin-A group (BoNT-A) consisted of three men and seven women (mean age 52.1 ± 11.4); and the onabotulinumtoxin-A and PT combined group (BoNT-A+PT) consisted of three men and seven women (mean age 51.3 ± 10.8).

### Onabotulinumtoxin-A Protocol (BoNT-A)

The Phase III Research Evaluating Migraine Prophylaxis Therapy (PREEMPT) protocol, used by numerous researchers for onabotulinumtoxin-A treatment (12), was applied. Said protocol is Food and Drug Administration (FDA)-approved, and it consists of 31 infiltrations with a small insulin needle. Each infiltration contains 5 units of onabotulinumtoxin-A for a total of 155 infiltrated units. The fixed infiltration sites are bilaterally located in various head muscles, namely, frontalis (20 U 4 sites), corrugator (10 U 2 sites), procerus (5 U 1 site), occipitalis (30 U 6 sites), temporalis (40 U 8 sites), trapezius (30 U 6 sites), and cervical paraspinal muscle group (20 U 4 sites). Injections were guided by electromyography and carried out by two neurologists in a single 40-min session.

### PT Protocol

The PT protocol was an integrated treatment, such as manual therapy (30 min) and active exercises (30 min). A total of 15 1-h individual sessions were carried out by two expert physiotherapists. Since extensive research identifies dysfunctions throughout the spine in patients with chronic migraine (28, 29), the manual therapy protocol started far from the trigeminal area, proceeding as follows: sacral area, diaphragm, dorsal and cervical spine, and cranial fascia (sub-occipital muscles and epicranial aponeurosis) (30, 31). With respect to active exercise, graded exercises were used to increase trunk performance, such as verticality function and endurance, during prolonged sitting positions. Exercises started from supine to sitting position on a balance board, where patients had to maintain trunk verticality combined with various head and upper and lower limb movements (16, 31).

### Onabotulinumtoxin-A and PT Combined Protocol

The onabotulinumtoxin-A and PT combined group underwent first the onabotulinumtoxin-A protocol and then, after 4 days, the PT protocol.

The final visit was programmed 3 months after the last treatment for each group (T2): headache parameters reported in the diary were analyzed, and PPT was re-evaluated following the same protocol as in T1. In the onabotulinumtoxin-A and onabotulinumtoxin-A+PT groups, the final visit was performed 3 months after the first cycle of onabotulinumtoxin-A. Each group (onabotulinumtoxin-A and onabotulinumtoxin-A+PT) underwent a single cycle of PREEMPT protocol.

### Statistical Analysis

Data were collected and analyzed with excel for the first analysis of the variance. Then, GraphPad InStat 3.06 was used for the statistical significance level was α 95% (0.05). Wilcoxon non-parametric test for a sample pair was used to compare T1 and T2 within treatments, while Kruskal-Wallis test (non-parametric ANOVA) was used for establishing the differences among treatment groups at T0 and T2. After non-parametric ANOVA, we used post–non-parametric Dunn's Multiple Comparison Test and the Bonferroni correction. The data graphic representation was performed with GraphPad Prism 8.4.1 (676).

## Results

No differences were found at Kruskal-Wallis test among the three groups at the first assessment (T1) in relation to age (*p* = 0.8), frequency of attacks (*p* = 0.08), duration of attacks (*p* = 0.08), pain intensity (*p* = 0.1), PPT over trapezius right (*p* = 0.3) and left (*p* = 0.4), levator scapulae right (*p* = 0.5) and left (*p* = 0.2), temporalis right (*p* = 0.07) and left (*p* = 0.1), sub-occipitalis right (*p* = 0.1) and left (*p* = 0.1), scalenus medius right (*p* = 0.2) and left (*p* = 0.1), and tensor fasciae latae right (*p* = 0.4) and left (*p* = 0.3). No patient reported intense sports activities (only walks on headache-free days); smoking; and adverse events. All patients reported a varied and balanced diet. All patients presented unilateral without fixed location.

### Pressure Pain Threshold

**Figure 1:**
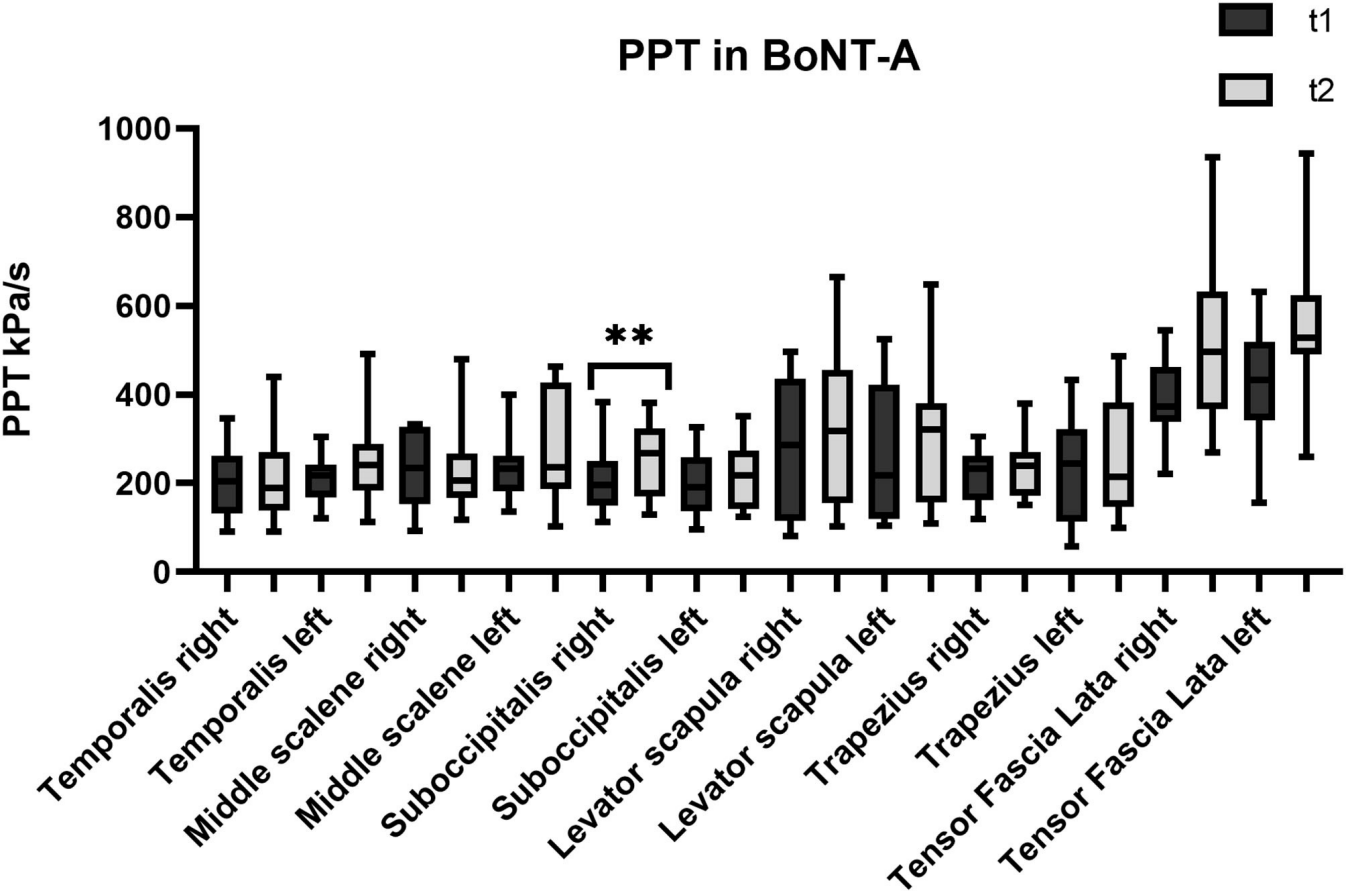
Pressure pain threshold (PPT) over the trigeminal and extra-trigeminal area before (T1) and after (T2) onabotulinumtoxin-A (BoNT-A) treatment. *p value < 0.01.

**Figure 2:**
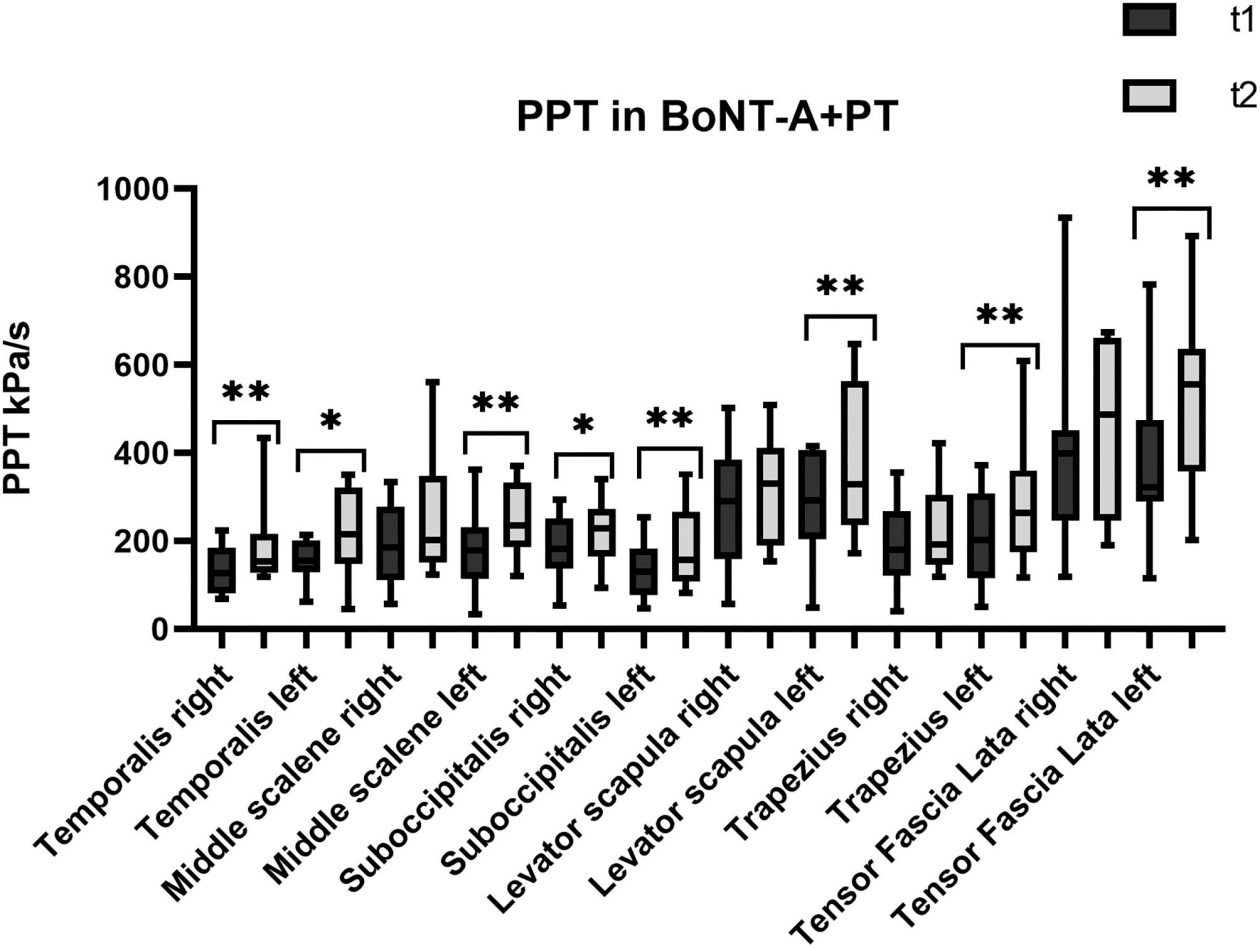
Pressure pain threshold over the trigeminal and extra-trigeminal area before (T1) and after (T2) onabotulinumtoxin-A+physical therapy (BoNT-A+PT) treatment. *p value < 0.05, **p value < 0.01.

**Figure 3:**
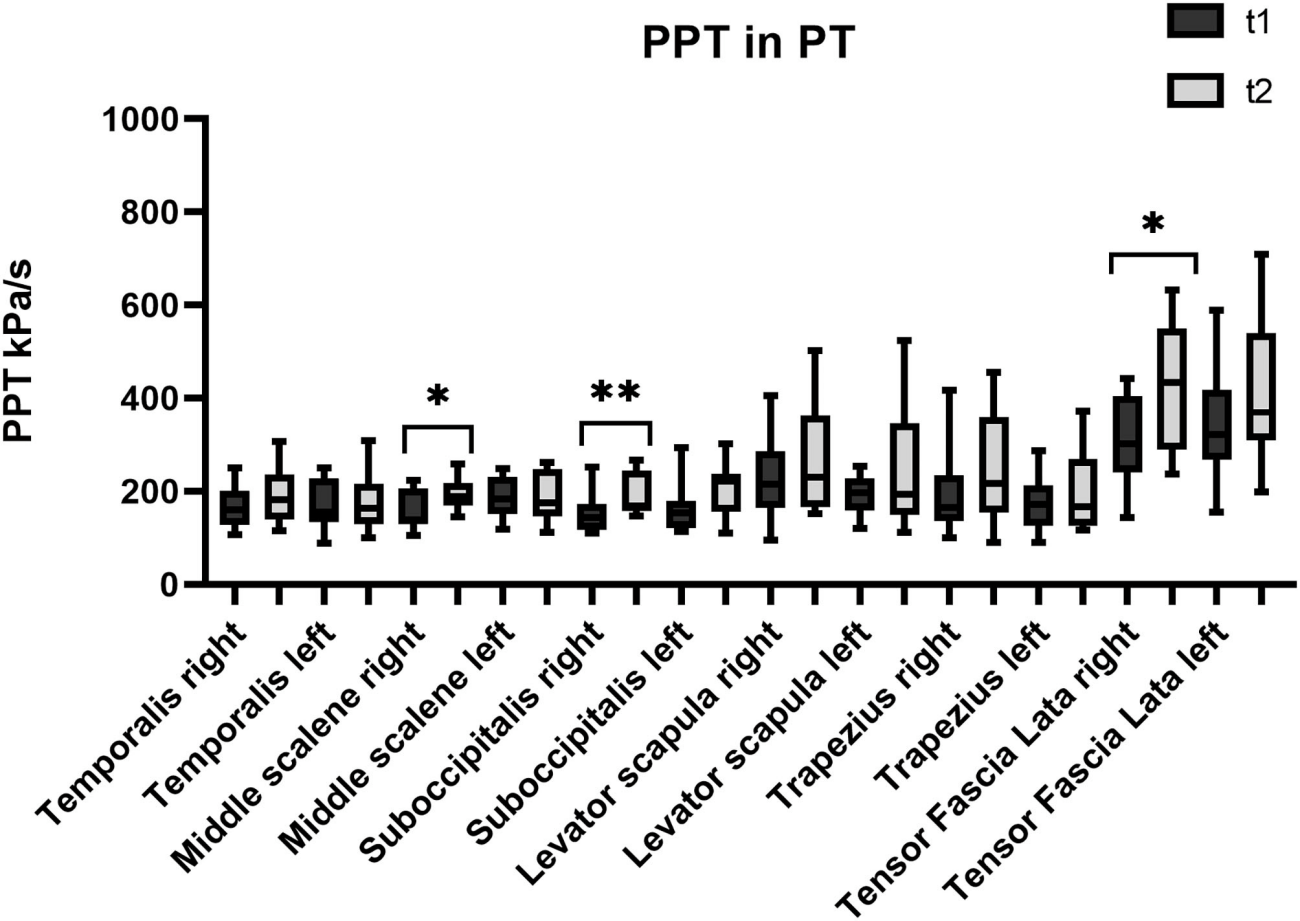
Pressure pain threshold over the trigeminal and extra-trigeminal area before (T1) and after (T2) physical therapy (PT) treatment.

The first set of analyses examined the impact of the three different treatments on the patients' PPT in the trigeminal area and farther from it. Table 1 presents an overview of the PPT in all muscles before and after each treatment. Figures 1–3 show that PPT values were improved significantly in more muscles during the combined onabotulinumtoxin-A and PT treatment (BoNT-A+PT) compared to the two mono-therapies (BoNT-A or PT only).

**Table 1:** Pressure pain threshold in onabotulinumtoxin-A (BoNT-A) group, in onabotulinumtoxin-A plus physical therapy group (BoNT-A+PT), and physical therapy group, before and after each treatment (at T1 and T2).

**Pressure pain threshold**	**BoNT-A median (IQR)**	**BoNT-A+PT median (IQR)**	**PT median (IQR)**
Temporalis left	t1 217.3 (167.9–222.5)	t1 155.8 (136.4–196.6)^*^	t1 155.4 (138.7–206.7)
	t2 241 (209.8–273.7)	t2 215.5 (154.8–300.2)	t2 162.6 (134–192.8)
Temporalis right	t1 204.1 (139.2–243.1)	t1 126.7 (83.9–169.8)^**^	t1 160.3 (133.2–184.9)
	t2 189.4 (146–252.8)	t2 153.2 (131.4–205.3)	t2 181.6 (145–222.1)
Sub-occipitalis left	t1 191.6 (141.5–253.7)	t1 130.3 (90.9–161.8)^**^	t1 154.1 (127.4–164)
	t2 217.2 (148.2–262.1)	t2 156.8 (117.7–241.2)	t2 222.1 (162.6–232.8)
Sub-occipitalis right	t1 196.2 (161–245.6)^**^	t1 180.8 (161–228.4)^*^	t1 144 (123.8–163.2)^**^
	t2 268.2 (190.2–306.4)	t2 229 (187.8–262.9)	t2 167.8 (158.7–224.3)
Middle scalene left	t1 233.3 (196.9–257.4)	t1 177.8 (125.4–213.3)^**^	t1 182.9 (158.6–226.8)
	t2 235.1 (211–392.1)	t2 235.2 (201.3–309.4)	t2 175.1 (156.9–229.1)
Middle scalene right	t1 234.5 (157–323.4)	t1 184.5 (133.2–243.6)	t1 136.2 (132.3–190.5)^*^
	t2 205.9 (185.7–241.3)	t2 202 (165.4–296.1)	t2 188 (176.3–216.8)
Trapezius left	t1 244.1 (133.8–304.7)	t1 200.8 (132.3–284.2)^*^	t1 172.4 (138.6–201)
	t2 213.6 (159.4–336.8)	t2 262.6 (195.8–345.9)	t2 165.9 (133.9–229.6)
Trapezius right	t1 233 (179.6–251.6)	t1 179.7 (130.6–244.9)	t1 165.2 (149–210.6)
	t2 238.4 (182.1–246.4)	t2 191.2 (154.7–280.9)	t2 216.6 (175.2–323.2)
Levator scapula left	t1 218.15 (123.4–383.8)	t1 291.6 (239.5–389.8)^*^	t1 196.9 (169.3–220.9)
	t2 322.1 (194.4–368.1)	t2 328.1 (260.3–520)	t2 193.6 (163.4–316.3)
Levator scapula right	t1 285.4 (124.9–418.1)	t1 290.7 (215.4–363.2)	t1 215.5 (175.4–255.4)
	t2 317.8 (178.2–421.5)	t2 318.6 (SD ± 129.3)	t2 229.6 (169.5–337.9)
Tensor fascia lata left	t1 434.1 (390.2–495.5)	t1 322.3 (314.9–425.9)^**^	t1 322.1 (307.3–399.1)
	t2 529 (504.6–570.7)	t2 555.8 (393.4–591.8)	t2 369.4 (321.7–473.1)
Tensor fascia lata right	t1 373 (349.2–433.9)	t1 398.5 (299–404.4)	t1 302.15 (260.3–387.8)^*^
	t2 496.8 (389.7–535.8)	t2 486.9 (277.4–655.3)	t2 433.7 (311.7–546.5)

* *p < 0.05*;

** *p < 0.01*;

*** *p < 0.001; Wilcoxon non-parametric test at the fist evaluation (T1) and at the end of each treatment (T2): BoNT-A, onabotulinumtoxin-A; BoNT-A+PT, onabotulinumtoxin-A plus physical therapy; PT, physical therapy*.

#### Temporalis Muscles

Regarding the temporalis muscles, PPT values show a significant increase only in the combined treatment BoNT-A+PT, both on the right (*p* = 0.003; CI_95%_ −107.6 to −8.8) and on the left side (*p* = 0.04; CI_95%_ −124.6 to −1.6). However, the Kruskal-Wallis test showed no significant differences among groups, neither for the right (*p* = 0.5) nor for the left temporalis (*p* = 0.1).

#### Sub-occipitalis

As regards the sub-occipitalis muscle, the combined treatment BoNT-A+PT group showed a statistically significant difference in PPT values, both on the right (*p* = 0.04; CI_95%_ −66.8 to −0.9) and on the left side (*p* = 0.009; CI_95%_ −73.8 to −18.2). In addition, PPT in sub-occipitalis was improved significantly, yet only for the right side, and only in the BoNT-A (*p* = 0.003; CI_95%_ −80.5 to −21.3) and in the PT (*p* = 0.003; CI_95%_ −65.4 to −11.7) treatment condition groups. The Kruskal-Wallis test did not show any significant difference among groups as regards the sub-occipitalis right (*p* = 0.1) and left (*p* = 0.5).

#### Scalenus Medius

Pressure pain threshold in the right scalenus medius was improved significantly only in the PT patients (*p* = 0.03; CI_95%_ −73.2 to −2.1), while the BoNT-A+PT combined treatment resulted in a significant difference in PPT in the left scalenus medius (*p* = 0.009; CI_95%_ −111.9 to −17.7). However, the Kruskal-Wallis test showed no significant difference among the groups as regards the right (*p* = 0.8) or the left scalenus medius (*p* = 0.1).

#### Trapezius

As regards the trapezius, a significant change in PPT was recorded in the combined treatment BoNT-A+PT group as regards the left side only (*p* = 0.03; CI_95%_ −150.3 to 7.8). The Kruskal-Wallis test did not show significant differences among the three treatment groups for either the right (*p* = 0.8) or the left side (*p* = 0.4) at the final evaluation.

#### Levator Scapulae

Similar to the trapezius, PPT values changed significantly in the BoNT-A+PT combined treatment group (*p* = 0.01; CI_95%_ −186.6 to −17.3) in the case of the left levator scapulae only. The Kruskal-Wallis test did not reveal any significant difference among the three groups for the levator scapulae, either right (*p* = 0.6) or left (*p* = 0.1).

#### Tensor Fasciae Latae

Finally, PPT values for the tensor fasciae latae registered an increase on the right side only in the PT group (*p* = 0.04; CI_95%_ −231.9 to −1.3) and on the left side only in the BoNT-A+PT combined treatment (*p* = 0.005; CI_95%_ −222.5 to −44.8). However, the Kruskal-Wallis test did not show significant differences between the three groups as regards the tensor fasciae latae, either right (*p* = 0.7) or left (*p* = 0.1).

### Headache Parameters

The following section illustrates the impact of the three treatments on the headache parameters, namely, migraine frequency, attack duration, and pain intensity.

Migraine frequency decreased significantly in all groups: in the BoNT-A group from a median of 19.5 (interquartile range [IQR] 19–24.5) days to a median of 12.5 days (IQR 9–22.75) (*p* = 0.007, CI_95%_ 1.7–10.2); in the BoNT-A+PT group from a median of 29 (IQR 22–30) days to a median of 19.5 (IQR 16–23) days (*p* = 0.003, CI_95%_ 4.5–9.8); in the PT group from a median of 17.5 (IQR 16–24.7) days to a median of 11.5 (IQR 5.7–18) days (*p* = 0.002, CI_95%_ 6–4.1). The Kruskal-Wallis test did not show significant differences among groups as regards frequency reduction (*p* = 0.1). Moreover, the duration of attacks was decreased significantly in all groups: in the BoNT-A group from a median of 132 (IQR 86.5–303.5) h to a median of 52 (27–145) h (*p* = 0.009, CI_95%_ 19.9–190.4); in the BoNT-A+PT group from a median of 160 (IQR 119.2–484.5) h to a median of 102.5 (IQR 43.25–201) h (*p* = 0.002, CI_95%_ 13.3–290.6); in the PT group from a median of 91.5 (IQR 58–130.5) h to a median of 58 (IQR 21–87) h (*p* = 0.009, CI_95%_ 11.2–57.5). No statistically significant difference among groups was found regarding this parameter (*p* = 0.2). The only statistically significant difference in pain intensity was observed in the BoNT-A (*p* = 0.01; CI_95%_ +0.8/+3.1) and in the BoNT-A+PT groups (*p* = 0.007; CI_95%_ +0.8/+3.1). Indeed, the intensity of pain assessed with the numeric pain rating scale (0–10) was decreased from a median of 8 (IQR 7.25–8) to a median of 5.5 (IQR 5–6.7) in the BoNT-A patients, from a median of 8 (IQR 7–8) to a median of 6 (IQR 5–6.7) in the BoNT-A+PT patients, and from a median of 7 (IQR 6–7.7) to 6.5 (IQR 5–7) in the PT patients. Although the Kruskal-Wallis test did not record a significant difference between the groups as regards this parameter (*p* = 0.9) (Supplementary Table 1).

## Discussion

Previous studies highlighted the importance of assessing the PPT as a clinical outcome of trigeminal system sensitization in patients with chronic migraine (8–10). Our study investigated the effect of different treatments on PPT, namely, (a) onabotulinumtoxin-A (BoNT-A) only, (b) PT (PT) only, and (c) onabotulinumtoxin-A and PT combined (BoNT-A+PT). A first interesting finding was that PPT values were improved significantly in a higher number of muscles in the combined treatment group (BoNT-A+PT) compared to the other two groups. The frequency and the duration of headache attacks were reduced significantly in all groups, with the BoNT-A and the BoNT-A+PT groups scoring better than the PT group as regards pain intensity. Moreover, the BoNT-A+PT combined treatment proved more helpful in reducing pain intensity than the BoNT-A treatment. Perhaps the most interesting finding was that the combined BoNT-A+PT treatment showed statistically significant differences both in PPT and in all other headache parameters.

As regards PPT, all three treatments resulted in its increase in almost all muscles, but there were differences between the groups: the combined treatment BoNT-A+PT produced a significant improvement of eight out of 12 points; the PT treatment registered an improvement only in three out of 12 points; and the BoNT-A group resulted in an increase in only one out of 12 points. These parameters have never been studied in patients with chronic migraine after the BoNT-A, the PT, or the combined treatments. Previous studies have reported non-significant changes in pain intensity and range of motion in the head and neck, after the BoNT-A treatment (31, 32). Our results appear to be consistent, with PPT values increasing in general in several muscles, although the increase was significant in only one of them. A possible explanation for this might be that an individualized BoNT-A protocol is more useful in pericranial myofascial pain linked to migraine than in the fixed sites of the PREEMPT protocol (33). With respect to the PT treatment, studies show moderate evidence that manual therapy may increase PPT values with a local and widespread analgesic effect (34, 35). Our results appear to be consistent, with PPT values rising in general in several muscles, although this increase was significant in only three of them, i.e., two muscles in the trigeminal area (sub-occipitalis right and middle scalene right) and one outside said area (tensor fasciae latae right). As regards the combined BoNT-A+PT treatment, only one study in the relevant literature describes the positive effect of physiotherapy combined with medication (topiramate and amitriptyline) on cervical PPT in patients with migraine (36). However, our most relevant clinical finding was that PPT values increased bilaterally in temporalis and sub-occipitalis muscles in patients that were administered the BoNT-A+PT combined treatment. These two muscles are the most assessed and treated in migraine since they appear to play a pivotal role in headache due to their anatomical connections (8, 10, 13, 14, 17, 37). The temporalis is innerved by the trigeminal nerve and the sub-occipitalis is innerved by the C1 and by the greater occipital nerve. Furthermore, the sub-occipitalis (particularly rectus capitis posterior) has an anatomic link with the dura mater, which is innerved by the ophthalmic division of the trigeminal nerve and by the greater occipital nerve (38, 39). This result may suggest that the BoNT-A+PT combined treatment may result in a higher pain-modulation effect than mono-therapy on this migraine-interested area.

As regards the cervical area from C1 to C4, we found a significant improvement in PPT in the trapezius, levator scapulae, and left scalenus medius. We hypothesized that the PT in addition to the BoNT-A may facilitate the release of tissue contractions in the upper cervical spine, which, in turn, could lead to increased craniocervical PPT (30, 31, 36). The tensor fasciae latae muscle was the area outside of the trigeminal system chosen to assess the effect of the widespread pain. Significant differences were found only on one side in both the BoNT-A+PT combined treatment and in the PT treatment groups. We suppose that an integrated physiotherapy treatment (manual therapy and active exercises) might have an additional desensitization effect throughout the body.

The second set of questions in our study sought to determine the effect of the three treatments on the headache parameters. The results are in line with those of Lemmens (16, 40), which prove that active exercises are as effective as pharmacological treatments as regards attack frequency and duration but not as regards pain intensity. Indeed, in our study, pain intensity decreased only in the BoNT-A and in the BoNT-A+PT groups. This supports our hypothesis that BoNT-A+PT combined treatment may be more effective than the BoNT-A treatment alone in tackling pain intensity (31, 41).

Concerning limitations, first, the absence of randomization would risk the possibility of a placebo/nocebo effect and would risk the possibility to not remark the differences due to the treatments. However, our study is an observational study and it evaluates the normal clinical practice that is based on the patient's choice and specialist's opinion. Second, a follow-up study and more PPT assessments during treatments would be helpful to highlight the differences among the different therapies over time. Indeed, many studies show that repetitive cycles of onabotulinumtoxin-A treatment over 1 year could lead to better outcomes in chronic migraine patients (13, 14, 42, 43). Conversely, repetitive cycles of BoNT-A+PT could lead to prolonged efficacy in terms of headache parameters and PPT. Third, consumption of symptomatic medication may be an uncontrolled variable. For this reason, according to the International Headache Society (19), we asked patients to limit the consumption to two times a week at most and to report both dosage and frequency of consumption in their headache diary. Fourth, gender differences may be a variable. For that reason, we assessed the PPT in female patients in the late follicular phase exclusively. Fifth, the small sample. The decision to include a small sample of participants depended on the rigorous methodology in terms of inclusion criteria, algometric assessments (Andersen's Systematic Review Guidelines), and collection of headache parameters. These restricted criteria did not allow us to recruit a larger sample. Despite that, the present study has three strong points: first, it is the first study investigating the effects on PPT of three treatments with a specific protocol in patients with migraine; second, it uses the guidelines of Andersen's systematic review for standardization of PPT assessment in patients with migraine; third, it assesses PPT not only within the trigeminal complex but also far from that area in patients with migraine.

Our findings, while preliminary, suggest several practical implications. First, the BoNT-A and the PT treatments together may exert a desensitization effect not only on the trigeminal area but also throughout the body. The sole BoNT-A treatment is more effective in tackling pain intensity than the PT treatment alone, due to the inhibition of neurogenic inflammation and to the reduction of CGRP expression. (12). Furthermore, an individualized protocol and repetitive cycles of injections may be more effective in improving PPT values than a single injection of PREEMPT protocol (13, 14, 33, 42, 43). On the other hand, the PT treatment with manual therapy reduces neural inflammation, while resulting in changes in tissue pathology and activation of the primary nociceptive afferents (17, 44). The PT treatment with active exercises affects pain modulation through the activation of the endogenous opioid system that could lead to reduced brain excitability and chronic muscle hyperalgesia (7, 16, 40). Consequently, the combined treatment, such as the BoNT-A treatment and an integrated protocol of PT, promotes additional improvement in the clinical management of chronic migraine. A combination of pharmacological and non-pharmacological treatments enhances analgesic effects on headache parameters and widespread hyperalgesia in patients with chronic migraine.

In conclusion, our observational cohort study suggests that a combined multi-disciplinary approach has additional effects on pain modulation in patients with chronic migraine, with respect to the two treatments administered individually. A randomized controlled trial could be useful to support our findings by offering a correlation between PPT values and headache parameters and by showing differences between male and female patients.

## Data Availability Statement

The raw data supporting the conclusions of this article will be made available by the authors, without undue reservation.

## Ethics Statement

The studies involving human participants were reviewed and approved by Comitato Etico Unico Regionale (CEUR); project identification code 143_2018; ID 2432. The patients/participants provided their written informed consent to participate in this study.

## Author Contributions

MD, AGr, and PM contributed to the conception and design of the study. MD and MC performed the algometer assessment and wrote the first draft of the manuscript. AGa and MC organized the database. MD and AGa performed the statistical analysis. MD, AGr, MC, AGa, and PM wrote sections of the manuscript. All authors contributed to manuscript revision, read, and approved the submitted version.

## Conflict of Interest

The authors declare that the research was conducted in the absence of any commercial or financial relationships that could be construed as a potential conflict of interest.

## Publisher's Note

All claims expressed in this article are solely those of the authors and do not necessarily represent those of their affiliated organizations, or those of the publisher, the editors and the reviewers. Any product that may be evaluated in this article, or claim that may be made by its manufacturer, is not guaranteed or endorsed by the publisher.

## Abbreviations

PT: Physiotherapy
BoNT: A Onabolulinumtoxin-A
PPT: Pressure Pain Threshold.
