# Supplementary material for: Algometer Assessment of Pressure Pain Threshold After Onabotulinumtoxin-A and Physical Therapy Treatments in Patients With Chronic Migraine: An Observational Study
Source: Front Pain Res (Lausanne). 2022 Feb 10;3:770397. doi: 10.3389/fpain.2022.770397 (PMC8915742; doi:10.3389/fpain.2022.770397)
Supplement: Supplementary file 1 [file Table_1.docx]

Supplementary Material

# Supplementary Tables

Table 1. Headache Parameters in Onabotulinumtoxin-A (BoNT-A) group, in Onabotulinumtoxin-A plus Physical Therapy group (BoNT-A+PT) and in Physical Therapy group, before and after each treatment (at T1 and T2).

| Headache parameters | BoNT-A | BoNT-A+PT | PT |
| --- | --- | --- | --- |
| Frequency t1 | 21.5(SD±5.2)** | 25.9(SD±6)** | 20.4(SD±5.5)** |
| t2 | 15.5(SD±8.4) | 18.7(SD±7.4) | 12.6(SD±8.4) |
| Duration t1 | 197.7(SD±174)** | 301.8(SD±260.6)** | 108.5(SD±77.6)** |
| t2 | 92.5(SD±94.2) | 149.8(SD±141.3) | 74.1(SD±73.1 ) |
| Pain Intensity t1 | 7.7(SD±0.8) | 7.6(SD±0.5) | 6.8(SD±1.3) |
| t2 | 5.7(SD±1.6)* | 5.6(SD±1.6)** | 5.8(SD±1.3) |

*p<0.05; **p<0.01 Wilcoxon non-parametric test at the fist evaluation (t1) and at the end of each treatment (t2): Onabotulunimtoxin-A (BoNT-A); Onabotulinumtoxin-A plus Physical Therapy (BoNT-A+PT); Physical Tharapy (PT).
