# Supplementary material for: Algometer Assessment of Pressure Pain Threshold After Onabotulinumtoxin-A and Physical Therapy Treatments in Patients With Chronic Migraine: An Observational Study
Source: Front Pain Res (Lausanne). 2022 Feb 10;3:770397. doi: 10.3389/fpain.2022.770397 (PMC8915742; doi:10.3389/fpain.2022.770397)
Supplement: Supplementary file 2 [file Data_Sheet_1.DOCX]

**BESELINE AGE**

Kruskal-Wallis Test (Nonparametric ANOVA)

The P value is 0.8841, considered not significant.

Variation among column medians is not significantly greater than expected by chance.

The P value is approximate (from chi-square distribution) because

at least one column has two or more identical values.

Calculation detail

Number Sum Mean

of of of

Group Points Ranks Ranks

=============== ======= ======= =======

b 10 164.50 16.450

b+pt 10 145.00 14.500

pt 10 155.50 15.550

Kruskal-Wallis Statistic KW = 0.2463 (corrected for ties)

Post tests were not calculated because the P value was greater

than 0.05.

Summary of Data

Number

of

Group Points Median Minimum Maximum

=============== ====== ======== ======== ========

b 10 53.500 40.000 79.000

b+ 10 48.000 39.000 69.000

pt 10 51.500 24.000 76.000

**BASELINE TEMPORALIS right**

Kruskal-Wallis Test (Nonparametric ANOVA)

The P value is 0.0663, considered not quite significant.

Variation among column medians is not significantly greater than expected by chance.

The P value is approximate (from chi-square distribution) because

at least one column has two or more identical values.

Calculation detail

Number Sum Mean

of of of

Group Points Ranks Ranks

=============== ======= ======= =======

b 10 196.00 19.600

b+pt 10 105.50 10.550

pt 10 163.50 16.350

Kruskal-Wallis Statistic KW = 5.426 (corrected for ties)

Post tests were not calculated because the P value was greater

than 0.05.

Summary of Data

Number

of

Group Points Median Minimum Maximum

=============== ====== ======== ======== ========

b 10 204.10 90.100 346.60

b+pt 10 126.70 67.900 222.80

pt 10 160.35 106.50 249.50

**Temporalis right only botox t1 vs t2**

Wilcoxon matched-pairs signed-ranks test

Does the median of the differences between b t1 and b t2 differ signficantly from zero?

The two-tailed P value is 0.5566, considered not significant.

Calculation details

Sum of all signed ranks (W) = -13.000

Sum of positive ranks (T+) = 21.000

Sum of negative ranks (T-) = -34.000

Number of pairs = 10

Assumption test: Was the pairing effective?

Nonparametric Spearman correlation coefficient (r) = 0.4061

The one-tailed P value is 0.1237, considered not significant.

Effective pairing results in a significant correlation between the

columns. With these data, the pairing (or matching) appears

not to be effective. The unpaired test may be more appropriate.

Summary of Data

Parameter: b t1 b t2 Difference

Mean: 199.66 216.32 -16.660

# of points: 10 10 10

Std deviation: 81.915 103.17 114.90

Std error: 25.904 32.624 36.335

Minimum: 90.100 90.800 -224.10

Maximum: 346.60 440.30 182.60

Median: 204.10 189.40 -34.550

Lower 95% CI: 141.07 142.52 -98.849

Upper 95% CI: 258.25 290.12 65.529

**BOTOX +PT TEMPORALIS right T1 VS T2**

Wilcoxon matched-pairs signed-ranks test

Does the median of the differences between Column A and Column B differ signficantly from zero?

The two-tailed P value is 0.0039, considered very significant.

Calculation details

Sum of all signed ranks (W) = -45.000

Sum of positive ranks (T+) = 0.000

Sum of negative ranks (T-) = -45.000

Number of pairs = 9

Note: 1 pair was excluded from calculations because both

values were equal.

Assumption test: Was the pairing effective?

Nonparametric Spearman correlation coefficient (r) = 0.6098

The one-tailed P value is 0.0334, considered significant.

Effective pairing results in a significant correlation between the

columns. With these data, the pairing (or matching) appears

not to be effective. The unpaired test may be more appropriate.

Summary of Data

Parameter: B+PT t1 B+PT t2 Difference

Mean: 130.30 188.58 -58.280

# of points: 10 10 10

Std deviation: 52.785 95.812 69.076

Std error: 16.692 30.298 21.844

Minimum: 67.900 118.20 -211.70

Maximum: 222.80 434.50 0.000

Median: 126.70 153.20 -36.600

Lower 95% CI: 92.543 120.05 -107.69

Upper 95% CI: 168.06 257.11 -8.869

**PT ONLY TEMPORALALIS right T1 vs T2**

Wilcoxon matched-pairs signed-ranks test

Does the median of the differences between FT V1 and FT V2 differ signficantly from zero?

The two-tailed P value is 0.1309, considered not significant.

Calculation details

Sum of all signed ranks (W) = -31.000

Sum of positive ranks (T+) = 12.000

Sum of negative ranks (T-) = -43.000

Number of pairs = 10

Assumption test: Was the pairing effective?

Nonparametric Spearman correlation coefficient (r) = 0.6364

The one-tailed P value is 0.0272, considered significant.

Effective pairing results in a significant correlation between the

columns. With these data, the pairing (or matching) appears

not to be effective. The unpaired test may be more appropriate.

Summary of Data

Parameter: PT T1 PT T2 Difference

Mean: 163.76 193.43 -29.670

# of points: 10 10 10

Std deviation: 46.590 63.243 51.087

Std error: 14.733 19.999 16.155

Minimum: 106.50 114.30 -144.90

Maximum: 249.50 306.40 29.300

Median: 160.35 181.60 -24.850

Lower 95% CI: 130.43 148.19 -66.213

Upper 95% CI: 197.09 238.67 6.873

**FINAL EVALUATION MULTIPLE COMPARISON TEST temporalis right among groups**

Kruskal-Wallis Test (Nonparametric ANOVA)

The P value is 0.5975, considered not significant.

Variation among column medians is not significantly greater than expected by chance.

The P value is approximate (from chi-square distribution) because

at least one column has two or more identical values.

Calculation detail

Number Sum Mean

of of of

Group Points Ranks Ranks

=============== ======= ======= =======

b 10 172.00 17.200

b+pt 10 133.00 13.300

pt 10 160.00 16.000

Kruskal-Wallis Statistic KW = 1.030 (corrected for ties)

Post tests were not calculated because the P value was greater

than 0.05.

Summary of Data

Number

of

Group Points Median Minimum Maximum

=============== ====== ======== ======== ========

b 10 189.40 90.800 440.30

b+pt 10 153.20 118.20 434.50

pt 10 181.60 114.30 306.40

**BONFERRONI CORRECTION OF FINAL EVALUATION MULTIPLE COMPARISON TEST temporalis left among groups**

One-way Analysis of Variance (ANOVA)

The P value is 0.7606, considered not significant.

Variation among column means is not significantly greater than expected

by chance.

Bonferroni Multiple Comparisons Test

If the value of t is greater than 2.552 then the P value is less

than 0.05.

Mean

Comparison Difference t P value

================================== ========== ======= ===========

b vs b+pt 27.740 0.6961 ns P>0.05

b vs pt 22.890 0.5744 ns P>0.05

b+pt vs pt -4.850 0.1217 ns P>0.05

Mean 95% Confidence Interval

Difference Difference From To

================================== ========== ======= =======

b - b+pt 27.740 -73.981 129.46

b - pt 22.890 -78.831 124.61

b+pt - pt -4.850 -106.57 96.871

Assumption test: Are the standard deviations of the groups equal?

ANOVA assumes that the data are sampled from populations with identical SDs. This assumption is tested using the method of Bartlett.

Bartlett statistic (corrected) = 2.127

The P value is 0.3453.

Bartlett's test suggests that the differences among the SDs is not significant.

Assumption test: Are the data sampled from Gaussian distributions?

ANOVA assumes that the data are sampled from populations that follow

Gaussian distributions. This assumption is tested using the method

Kolmogorov and Smirnov:

Group KS P Value Passed normality test?

=============== ====== ======== =======================

b 0.1712 >0.10 Yes

b+pt 0.2160 >0.10 Yes

pt 0.1338 >0.10 Yes

Intermediate calculations. ANOVA table

Source of Degrees of Sum of Mean

variation freedom squares square

============================ ========== ======== ========

Treatments (between columns) 2 4389.9 2195.0

Residuals (within columns) 27 214406 7941.0

---------------------------- ---------- --------

Total 29 218796

F = 0.2764 =(MStreatment/MSresidual)

Summary of Data

Number Standard

of Standard Error of

Group Points Mean Deviation Mean Median

=============== ====== ======== ========= ======== ========

b 10 216.32 103.17 32.624 189.40

b+pt 10 188.58 95.812 30.298 153.20

pt 10 193.43 63.243 19.999 181.60

95% Confidence Interval

Group Minimum Maximum From To

=============== ======== ======== ========== ==========

b 90.800 440.30 142.52 290.12

b+pt 118.20 434.50 120.05 257.11

pt 114.30 306.40 148.19 238.67

**BASELINE TEMPORALIS LEFT**

Kruskal-Wallis Test (Nonparametric ANOVA)

The P value is 0.1009, considered not significant.

Variation among column medians is not significantly greater than expected by chance.

The P value is approximate (from chi-square distribution) because at least one column has two or more identical values.

Calculation detail

Number Sum Mean

of of of

Group Points Ranks Ranks

=============== ======= ======= =======

b 10 203.00 20.300

b+pt 10 124.00 12.400

pt 10 138.00 13.800

Kruskal-Wallis Statistic KW = 4.587 (corrected for ties)

Post tests were not calculated because the P value was greater

than 0.05.

Summary of Data

Number

of

Group Points Median Minimum Maximum

=============== ====== ======== ======== ========

b 10 217.30 120.00 304.40

b+pt 10 155.80 62.000 213.00

pt 10 155.40 87.500 249.50

**BOTOX TEMPORALALIS LEFT T1vs T2**

Wilcoxon matched-pairs signed-ranks test

Does the median of the differences between b and b t2 differ signficantly from zero?

The two-tailed P value is 0.1602, considered not significant.

Calculation details

Sum of all signed ranks (W) = -29.000

Sum of positive ranks (T+) = 13.000

Sum of negative ranks (T-) = -42.000

Number of pairs = 10

Assumption test: Was the pairing effective?

Nonparametric Spearman correlation coefficient (r) = 0.5714

The one-tailed P value is 0.0441, considered significant.

Effective pairing results in a significant correlation between the

columns. With these data, the pairing (or matching) appears

not to be effective. The unpaired test may be more appropriate.

Summary of Data

Parameter: b b t2 Difference

Mean: 210.61 249.99 -39.380

# of points: 10 10 10

Std deviation: 58.394 105.75 96.761

Std error: 18.466 33.441 30.599

Minimum: 120.00 111.70 -270.80

Maximum: 304.40 491.90 108.50

Median: 217.30 241.05 -23.150

Lower 95% CI: 168.84 174.35 -108.59

Upper 95% CI: 252.38 325.63 29.834

**B+PT TEMPORALIS LEFT T1 VS T2**

Wilcoxon matched-pairs signed-ranks test

Does the median of the differences between Column A and Column B

differ signficantly from zero?

The two-tailed P value is **0.0488, considered significant.**

Calculation details

Sum of all signed ranks (W) = -39.000

Sum of positive ranks (T+) = 8.000

Sum of negative ranks (T-) = -47.000

Number of pairs = 10

Assumption test: Was the pairing effective?

Nonparametric Spearman correlation coefficient (r) = 0.5515

The one-tailed P value is 0.0524, considered not quite significant.

Effective pairing results in a significant correlation between the

columns. With these data, the pairing (or matching) appears

not to be effective. The unpaired test may be more appropriate.

Summary of Data

Parameter: B+PT B+PT Difference

Mean: 157.01 219.96 -62.950

# of points: 10 10 10

Std deviation: 47.801 96.886 85.713

Std error: 15.116 30.638 27.105

Minimum: 62.000 45.700 -173.70

Maximum: 213.00 350.00 88.900

Median: 155.80 215.50 -63.950

Lower 95% CI: 122.82 150.66 -124.26

Upper 95% CI: 191.20 289.26 -1.639

**PT TEMPORALIS LEFT T1 VS T2**

Wilcoxon matched-pairs signed-ranks test

Does the median of the differences between FT and FT differ signficantly from zero?

The two-tailed P value is 0.9219, considered not significant.

Calculation details

Sum of all signed ranks (W) = 3.000

Sum of positive ranks (T+) = 29.000

Sum of negative ranks (T-) = -26.000

Number of pairs = 10

Assumption test: Was the pairing effective?

Nonparametric Spearman correlation coefficient (r) = 0.2121

The one-tailed P value is 0.2801, considered not significant.

Effective pairing results in a significant correlation between the

columns. With these data, the pairing (or matching) appears

not to be effective. The unpaired test may be more appropriate.

Summary of Data

Parameter: PT PT Difference

Mean: 167.30 176.16 -8.860

# of points: 10 10 10

Std deviation: 53.348 66.063 75.519

Std error: 16.870 20.891 23.881

Minimum: 87.500 99.300 -180.20

Maximum: 249.50 307.60 75.700

Median: 155.40 162.65 4.100

Lower 95% CI: 129.14 128.90 -62.879

Upper 95% CI: 205.46 223.42 45.159

**FINAL EVALUATION TEMPORALIS LEFT AMONG GROUPS MULTIPLE COMPARISON**

Kruskal-Wallis Test (Nonparametric ANOVA)

The P value is 0.1996, considered not significant.

Variation among column medians is not significantly greater than expected by chance.

The P value is approximate (from chi-square distribution) because exact calculations would have taken too long.

Calculation detail

Number Sum Mean

of of of

Group Points Ranks Ranks

=============== ======= ======= =======

b 10 182.00 18.200

b+pt 10 168.00 16.800

pt 10 115.00 11.500

Kruskal-Wallis Statistic KW = 3.223

Post tests were not calculated because the P value was greater

than 0.05.

Summary of Data

Number

of

Group Points Median Minimum Maximum

=============== ====== ======== ======== ========

b 10 241.05 111.70 491.90

b+pt 10 215.50 45.700 350.00

pt 10 162.65 99.300 307.60

**BONFERRONI CORRECTION OF FINAL EVALUATION MULTIPLE COMPARISON TEST temporalis left among groups**

One-way Analysis of Variance (ANOVA)

The P value is 0.2092, considered not significant.

Variation among column means is not significantly greater than expected by chance.

Bonferroni Multiple Comparisons Test

If the value of t is greater than 2.552 then the P value is less than 0.05.

Mean

Comparison Difference t P value

================================== ========== ======= ===========

b vs b+pt 30.030 0.7365 ns P>0.05

b vs pt 73.830 1.811 ns P>0.05

b+pt vs pt 43.800 1.074 ns P>0.05

Mean 95% Confidence Interval

Difference Difference From To

================================== ========== ======= =======

b - b+pt 30.030 -74.037 134.10

b - pt 73.830 -30.237 177.90

b+pt - pt 43.800 -60.267 147.87

Assumption test: Are the standard deviations of the groups equal?

ANOVA assumes that the data are sampled from populations with identical SDs. This assumption is tested using the method of Bartlett.

Bartlett statistic (corrected) = 1.936

The P value is 0.3799.

Bartlett's test suggests that the differences among the SDs is not significant.

Assumption test: Are the data sampled from Gaussian distributions?

ANOVA assumes that the data are sampled from populations that follow

Gaussian distributions. This assumption is tested using the method

Kolmogorov and Smirnov:

Group KS P Value Passed normality test?

=============== ====== ======== =======================

b 0.1945 >0.10 Yes

b+pt 0.1412 >0.10 Yes

pt 0.2142 >0.10 Yes

Intermediate calculations. ANOVA table

Source of Degrees of Sum of Mean

variation freedom squares square

============================ ========== ======== ========

Treatments (between columns) 2 27570 13785

Residuals (within columns) 27 224409 8311.4

---------------------------- ---------- --------

Total 29 251979

F = 1.659 =(MStreatment/MSresidual)

Summary of Data

Number Standard

of Standard Error of

Group Points Mean Deviation Mean Median

=============== ====== ======== ========= ======== ========

b 10 249.99 105.75 33.441 241.05

b+pt 10 219.96 96.886 30.638 215.50

pt 10 176.16 66.063 20.891 162.65

95% Confidence Interval

Group Minimum Maximum From To

=============== ======== ======== ========== ==========

b 111.70 491.90 174.35 325.63

b+pt 45.700 350.00 150.66 289.26

pt 99.300 307.60 128.90 223.42

**BASELINE Scalenus medius right**

Kruskal-Wallis Test (Nonparametric ANOVA)

The P value is 0.2057, considered not significant.

Variation among column medians is not significantly greater than expected by chance.

The P value is approximate (from chi-square distribution) because

at least one column has two or more identical values.

Calculation detail

Number Sum Mean

of of of

Group Points Ranks Ranks

=============== ======= ======= =======

b 10 190.00 19.000

b+pt 10 155.00 15.500

pt 10 120.00 12.000

Kruskal-Wallis Statistic KW = 3.163 (corrected for ties)

Post tests were not calculated because the P value was greater than 0.05.

Summary of Data

Number

of

Group Points Median Minimum Maximum

=============== ====== ======== ======== ========

b 10 234.55 91.800 332.50

b+pt 10 184.50 56.200 332.50

pt 10 136.20 104.50 223.40

**BOTOX Scalenus medius right T1 VS T2**

Wilcoxon matched-pairs signed-ranks test

Does the median of the differences between b 1 and b 2 differ signficantly from zero?

The two-tailed P value is 0.9219, considered not significant.

Calculation details

Sum of all signed ranks (W) = -3.000

Sum of positive ranks (T+) = 26.000

Sum of negative ranks (T-) = -29.000

Number of pairs = 10

Assumption test: Was the pairing effective?

Nonparametric Spearman correlation coefficient (r) = 0.3091

The one-tailed P value is 0.1935, considered not significant.

Effective pairing results in a significant correlation between the

columns. With these data, the pairing (or matching) appears

not to be effective. The unpaired test may be more appropriate.

Summary of Data

Parameter: b 1 b 2 Difference

Mean: 233.56 229.05 4.510

# of points: 10 10 10

Std deviation: 92.702 104.82 107.01

Std error: 29.315 33.146 33.841

Minimum: 91.800 117.60 -152.40

Maximum: 332.50 479.50 212.40

Median: 234.55 205.90 -33.100

Lower 95% CI: 167.25 154.07 -72.038

Upper 95% CI: 299.87 304.03 81.058

**BOTOX+PT Scalenus medius right T1 VS T2**

Wilcoxon matched-pairs signed-ranks test

Does the median of the differences between Column A and Column B

differ signficantly from zero?

The two-tailed P value is 0.1309, considered not significant.

Calculation details

Sum of all signed ranks (W) = -31.000

Sum of positive ranks (T+) = 12.000

Sum of negative ranks (T-) = -43.000

Number of pairs = 10

Assumption test: Was the pairing effective?

Nonparametric Spearman correlation coefficient (r) = 0.7333

The one-tailed P value is 0.0101, considered significant.

Effective pairing results in a significant correlation between the

columns. With these data, the pairing (or matching) appears

to be effective.

Summary of Data

Parameter: t1 t2 Difference

Mean: 192.31 253.10 -60.790

# of points: 10 10 10

Std deviation: 92.564 139.52 110.62

Std error: 29.271 44.119 34.983

Minimum: 56.200 124.10 -235.10

Maximum: 332.50 561.20 129.90

Median: 184.50 202.05 -48.450

Lower 95% CI: 126.10 153.30 -139.92

Upper 95% CI: 258.52 352.90 18.341

**PT Scalenus medius right T1 VS T2**

Wilcoxon matched-pairs signed-ranks test

Does the median of the differences between Column A and Column B

differ signficantly from zero?

The two-tailed P value is 0.0371, considered significant.

Calculation details

Sum of all signed ranks (W) = -41.000

Sum of positive ranks (T+) = 7.000

Sum of negative ranks (T-) = -48.000

Number of pairs = 10

Assumption test: Was the pairing effective?

Nonparametric Spearman correlation coefficient (r) = -0.07903

The negative correlation coefficient indicates that the pairing or

matching was NOT effective.

Summary of Data

Parameter: t1 t2 Difference

Mean: 157.52 195.20 -37.680

# of points: 10 10 10

Std deviation: 42.035 33.079 49.666

Std error: 13.293 10.460 15.706

Minimum: 104.50 145.60 -129.40

Maximum: 223.40 258.70 13.800

Median: 136.20 188.05 -22.150

Lower 95% CI: 127.45 171.54 -73.206

Upper 95% CI: 187.59 218.86 -2.154

**FINAL EVALUATION MULTIPLE COMPARISON AMONG GROUPS Scalenus medius right**

Kruskal-Wallis Test (Nonparametric ANOVA)

The P value is 0.8780, considered not significant.

Variation among column medians is not significantly greater than expected by chance.

The P value is approximate (from chi-square distribution) because at least one column has two or more identical values.

Calculation detail

Number Sum Mean

of of of

Group Points Ranks Ranks

=============== ======= ======= =======

b 10 159.50 15.950

b+pt 10 162.00 16.200

pt 10 143.50 14.350

Kruskal-Wallis Statistic KW = 0.2601 (corrected for ties)

Post tests were not calculated because the P value was greater than 0.05.

Summary of Data

Number

of

Group Points Median Minimum Maximum

=============== ====== ======== ======== ========

b 10 205.90 117.60 479.50

b+pt 10 202.05 124.10 561.20

pt 10 188.05 145.60 258.70

**BONFERRONI CORRECTION OF FINAL EVALUATION MULTIPLE COMPARISON AMONG GROUPS Scalenus medius right**

One-way Analysis of Variance (ANOVA)

The P value is 0.4577, considered not significant.

Variation among column means is not significantly greater than expected by chance.

Bonferroni Multiple Comparisons Test

If the value of t is greater than 2.552 then the P value is less than 0.05.

Mean

Comparison Difference t P value

================================== ========== ======= ===========

b vs b+pt -24.050 0.5244 ns P>0.05

b vs pt 33.850 0.7381 ns P>0.05

b+pt vs pt 57.900 1.263 ns P>0.05

Mean 95% Confidence Interval

Difference Difference From To

================================== ========== ======= =======

b - b+pt -24.050 -141.10 93.004

b - pt 33.850 -83.204 150.90

b+pt - pt 57.900 -59.154 174.95

Assumption test: Are the standard deviations of the groups equal?

ANOVA assumes that the data are sampled from populations with identical

SDs. This assumption is tested using the method of Bartlett.

Bartlett statistic (corrected) = 13.750

The P value is 0.0010.

Bartlett's test suggests that the differences among the SDs is very significant.

Since ANOVA assumes populations with equal SDs, you should consider transforming your data (reciprocal or log) or selecting a nonparametric test.

Assumption test: Are the data sampled from Gaussian distributions?

ANOVA assumes that the data are sampled from populations that follow Gaussian distributions. This assumption is tested using the method

Kolmogorov and Smirnov:

Group KS P Value Passed normality test?

=============== ====== ======== =======================

b 0.2537 0.0674 Yes

b+pt 0.3262 0.0034 No

pt 0.1850 >0.10 Yes

At least one column failed the normality test with P<0.05.

Consider using a nonparametric test or transforming the data

(i.e. converting to logarithms or reciprocals).

Intermediate calculations. ANOVA table

Source of Degrees of Sum of Mean

variation freedom squares square

============================ ========== ======== ========

Treatments (between columns) 2 16922 8461.1

Residuals (within columns) 27 283913 10515

---------------------------- ---------- --------

Total 29 300835

F = 0.8046 =(MStreatment/MSresidual)

Summary of Data

Number Standard

of Standard Error of

Group Points Mean Deviation Mean Median

=============== ====== ======== ========= ======== ========

b 10 229.05 104.82 33.146 205.90

b+pt 10 253.10 139.52 44.119 202.05

pt 10 195.20 33.079 10.460 188.05

95% Confidence Interval

Group Minimum Maximum From To

=============== ======== ======== ========== ==========

b 117.60 479.50 154.07 304.03

b+pt 124.10 561.20 153.30 352.90

pt 145.60 258.70 171.54 218.86

**BASELINE** **Scalenus medius left**

Kruskal-Wallis Test (Nonparametric ANOVA)

The P value is 0.1409, considered not significant.

Variation among column medians is not significantly greater than expected by chance.

The P value is approximate (from chi-square distribution) because

at least one column has two or more identical values.

Calculation detail

Number Sum Mean

of of of

Group Points Ranks Ranks

=============== ======= ======= =======

b 10 199.50 19.950

b+pt 10 127.00 12.700

pt 10 138.50 13.850

Kruskal-Wallis Statistic KW = 3.919 (corrected for ties)

Post tests were not calculated because the P value was greater

than 0.05.

Summary of Data

Number

of

Group Points Median Minimum Maximum

=============== ====== ======== ======== ========

b 10 233.30 136.50 399.70

b+pt 10 177.80 33.300 362.60

pt 10 182.95 118.20 248.20

**BOTOX Scalenus medius left T1 VS T2**

Wilcoxon matched-pairs signed-ranks test

Does the median of the differences between b 1 and b 2 differ signficantly from zero?

The two-tailed P value is 0.2324, considered not significant.

Calculation details

Sum of all signed ranks (W) = -25.000

Sum of positive ranks (T+) = 15.000

Sum of negative ranks (T-) = -40.000

Number of pairs = 10

Assumption test: Was the pairing effective?

Nonparametric Spearman correlation coefficient (r) = 0.5636

The one-tailed P value is 0.0481, considered significant.

Effective pairing results in a significant correlation between the

columns. With these data, the pairing (or matching) appears

not to be effective. The unpaired test may be more appropriate.

Summary of Data

Parameter: b 1 b 2 Difference

Mean: 234.75 274.83 -40.076

# of points: 10 10 10

Std deviation: 71.323 129.80 105.39

Std error: 22.554 41.046 33.326

Minimum: 136.50 102.56 -207.50

Maximum: 399.70 462.50 143.24

Median: 233.30 235.15 -29.350

Lower 95% CI: 183.73 181.98 -115.46

Upper 95% CI: 285.77 367.67 35.308

**B+PT Scalenus medius left T1 VS T2**

Wilcoxon matched-pairs signed-ranks test

Does the median of the differences between Column A and Column B

differ signficantly from zero?

The two-tailed P value is **0.0098, considered very significant.**

Calculation details

Sum of all signed ranks (W) = -49.000

Sum of positive ranks (T+) = 3.000

Sum of negative ranks (T-) = -52.000

Number of pairs = 10

Assumption test: Was the pairing effective?

Nonparametric Spearman correlation coefficient (r) = 0.6364

The one-tailed P value is 0.0272, considered significant.

Effective pairing results in a significant correlation between the

columns. With these data, the pairing (or matching) appears

not to be effective. The unpaired test may be more appropriate.

Summary of Data

Parameter: t1 t2 Difference

Mean: 179.70 244.51 -64.814

# of points: 10 10 10

Std deviation: 90.998 84.985 65.855

Std error: 28.776 26.875 20.825

Minimum: 33.300 120.80 -187.30

Maximum: 362.60 370.80 8.500

Median: 177.80 235.25 -47.250

Lower 95% CI: 114.60 183.72 -111.92

Upper 95% CI: 244.79 305.30 -17.707

**PT Scalenus medius left T1 VS T2**

Wilcoxon matched-pairs signed-ranks test

Does the median of the differences between FT and FT differ signficantly from zero?

The two-tailed P value is 0.9219, considered not significant.

Calculation details

Sum of all signed ranks (W) = 3.000

Sum of positive ranks (T+) = 29.000

Sum of negative ranks (T-) = -26.000

Number of pairs = 10

Assumption test: Was the pairing effective?

Nonparametric Spearman correlation coefficient (r) = 0.1581

The one-tailed P value is 0.3284, considered not significant.

Effective pairing results in a significant correlation between the

columns. With these data, the pairing (or matching) appears

not to be effective. The unpaired test may be more appropriate.

Summary of Data

Parameter: PT t1 PT t2 Difference

Mean: 187.68 185.64 2.040

# of points: 10 10 10

Std deviation: 45.111 52.597 61.259

Std error: 14.265 16.633 19.372

Minimum: 118.20 112.30 -105.10

Maximum: 248.20 261.90 95.400

Median: 182.95 175.10 -8.150

Lower 95% CI: 155.41 148.02 -41.779

Upper 95% CI: 219.95 223.26 45.859

**Scalenus medius left final evaluation among groups**

Kruskal-Wallis Test (Nonparametric ANOVA)

The P value is 0.1924, considered not significant.

Variation among column medians is not significantly greater than expected by chance.

The P value is approximate (from chi-square distribution) because

at least one column has two or more identical values.

Calculation detail

Number Sum Mean

of of of

Group Points Ranks Ranks

=============== ======= ======= =======

b 10 179.50 17.950

b+pt 10 171.50 17.150

pt 10 114.00 11.400

Kruskal-Wallis Statistic KW = 3.296 (corrected for ties)

Post tests were not calculated because the P value was greater than 0.05.

Summary of Data

Number

of

Group Points Median Minimum Maximum

=============== ====== ======== ======== ========

b 10 235.15 102.56 462.50

b+pt 10 235.25 120.80 370.80

pt 10 175.10 112.30 261.90

**BONFERRONI CORRECTION Scalenus medius left final evaluation**

One-way Analysis of Variance (ANOVA)

The P value is 0.1197, considered not significant.

Variation among column means is not significantly greater than expected by chance.

Bonferroni Multiple Comparisons Test

If the value of t is greater than 2.552 then the P value is less than 0.05.

Mean

Comparison Difference t P value

================================== ========== ======= ===========

b vs b+pt 30.316 0.7167 ns P>0.05

b vs pt 89.186 2.109 ns P>0.05

b+pt vs pt 58.870 1.392 ns P>0.05

Mean 95% Confidence Interval

Difference Difference From To

================================== ========== ======= =======

b - b+pt 30.316 -77.647 138.28

b - pt 89.186 -18.777 197.15

b+pt - pt 58.870 -49.093 166.83

Assumption test: Are the standard deviations of the groups equal?

ANOVA assumes that the data are sampled from populations with identical

SDs. This assumption is tested using the method of Bartlett.

Bartlett statistic (corrected) = 6.471

The P value is 0.0393.

Bartlett's test suggests that the differences among the SDs is

significant.

Since ANOVA assumes populations with equal SDs, you should consider

transforming your data (reciprocal or log) or selecting a

nonparametric test.

Assumption test: Are the data sampled from Gaussian distributions?

ANOVA assumes that the data are sampled from populations that follow

Gaussian distributions. This assumption is tested using the method

Kolmogorov and Smirnov:

Group KS P Value Passed normality test?

=============== ====== ======== =======================

b 0.1752 >0.10 Yes

b+pt 0.1297 >0.10 Yes

pt 0.2306 >0.10 Yes

Intermediate calculations. ANOVA table

Source of Degrees of Sum of Mean

variation freedom squares square

============================ ========== ======== ========

Treatments (between columns) 2 41130 20565

Residuals (within columns) 27 241527 8945.5

---------------------------- ---------- --------

Total 29 282657

F = 2.299 =(MStreatment/MSresidual)

Summary of Data

Number Standard

of Standard Error of

Group Points Mean Deviation Mean Median

=============== ====== ======== ========= ======== ========

b 10 274.83 129.80 41.046 235.15

b+pt 10 244.51 84.985 26.875 235.25

pt 10 185.64 52.597 16.633 175.10

95% Confidence Interval

Group Minimum Maximum From To

=============== ======== ======== ========== ==========

b 102.56 462.50 181.98 367.67

b+pt 120.80 370.80 183.72 305.30

pt 112.30 261.90 148.02 223.26

**TENSOR FASCIAE LATAE right BASELINE**

Kruskal-Wallis Test (Nonparametric ANOVA)

The P value is 0.4024, considered not significant.

Variation among column medians is not significantly greater than expected by chance.

The P value is approximate (from chi-square distribution) because

at least one column has two or more identical values.

Calculation detail

Number Sum Mean

of of of

Group Points Ranks Ranks

=============== ======= ======= =======

B 10 173.00 17.300

B+PT 10 167.50 16.750

PT 10 124.50 12.450

Kruskal-Wallis Statistic KW = 1.821 (corrected for ties)

Post tests were not calculated because the P value was greater

than 0.05.

Summary of Data

Number

of

Group Points Median Minimum Maximum

=============== ====== ======== ======== ========

B 10 373.00 220.80 545.50

B+PT 10 398.50 117.60 934.80

PT 10 302.15 143.10 442.30

**TFL right BOTOX t1/t2**

Wilcoxon matched-pairs signed-ranks test

Does the median of the differences between B1 and B2 differ signficantly from zero?

The two-tailed P value is 0.0645, considered not quite significant.

Calculation details

Sum of all signed ranks (W) = -37.000

Sum of positive ranks (T+) = 9.000

Sum of negative ranks (T-) = -46.000

Number of pairs = 10

Assumption test: Was the pairing effective?

Nonparametric Spearman correlation coefficient (r) = -0.06667

The negative correlation coefficient indicates that the pairing or

matching was NOT effective.

Summary of Data

Parameter: B1 B2 Difference

Mean: 386.94 533.89 -146.95

# of points: 10 10 10

Std deviation: 92.595 218.74 240.75

Std error: 29.281 69.170 76.133

Minimum: 220.80 268.50 -714.00

Maximum: 545.50 934.80 134.60

Median: 373.00 496.85 -98.850

Lower 95% CI: 320.71 377.43 -319.16

Upper 95% CI: 453.17 690.35 25.262

**TFL right BOTOX+pT t1/t2**

Wilcoxon matched-pairs signed-ranks test

Does the median of the differences between Column A and Column B

differ signficantly from zero?

The two-tailed P value is 0.2324, considered not significant.

Calculation details

Sum of all signed ranks (W) = -25.000

Sum of positive ranks (T+) = 15.000

Sum of negative ranks (T-) = -40.000

Number of pairs = 10

Assumption test: Was the pairing effective?

Nonparametric Spearman correlation coefficient (r) = 0.9146

The one-tailed P value is 0.0003, considered extremely significant.

Effective pairing results in a significant correlation between the

columns. With these data, the pairing (or matching) appears

to be effective.

Summary of Data

Parameter: t1 t2 Difference

Mean: 401.60 465.82 -64.220

# of points: 10 10 10

Std deviation: 233.18 194.40 158.63

Std error: 73.737 61.475 50.163

Minimum: 117.60 190.70 -257.40

Maximum: 934.80 673.80 261.00

Median: 398.50 486.90 -93.600

Lower 95% CI: 234.81 326.76 -177.69

Upper 95% CI: 568.39 604.88 49.248

**TFL right pT T1 vs t2**

Wilcoxon matched-pairs signed-ranks test

Does the median of the differences between FT and FT differ signficantly from zero?

The two-tailed P value is 0.0488, considered significant.

Calculation details

Sum of all signed ranks (W) = -39.000

Sum of positive ranks (T+) = 8.000

Sum of negative ranks (T-) = -47.000

Number of pairs = 10

Assumption test: Was the pairing effective?

Nonparametric Spearman correlation coefficient (r) = 0.2242

The one-tailed P value is 0.2683, considered not significant.

Effective pairing results in a significant correlation between the

columns. With these data, the pairing (or matching) appears

not to be effective. The unpaired test may be more appropriate.

Summary of Data

Parameter: PT t1 PT t2 Difference

Mean: 311.44 428.09 -116.65

# of points: 10 10 10

Std deviation: 97.736 141.24 161.23

Std error: 30.907 44.665 50.987

Minimum: 143.10 236.50 -405.70

Maximum: 442.30 632.40 129.40

Median: 302.15 433.75 -148.60

Lower 95% CI: 241.53 327.06 -231.98

Upper 95% CI: 381.35 529.12 -1.318

**FINAL EVALUATION AMONG GROUPS TFL RIGHT**

Kruskal-Wallis Test (Nonparametric ANOVA)

The P value is 0.7159, considered not significant.

Variation among column medians is not significantly greater than expected by chance.

The P value is approximate (from chi-square distribution) because

at least one column has two or more identical values.

Calculation detail

Number Sum Mean

of of of

Group Points Ranks Ranks

=============== ======= ======= =======

B 10 168.00 16.800

B+PT 10 160.00 16.000

PT 10 137.00 13.700

Kruskal-Wallis Statistic KW = 0.6685 (corrected for ties)

Post tests were not calculated because the P value was greater

than 0.05.

Summary of Data

Number

of

Group Points Median Minimum Maximum

=============== ====== ======== ======== ========

B 10 496.85 268.50 934.80

B+PT 10 486.90 190.70 673.80

PT 10 433.75 236.50 632.40

**BONFERRONI CORRECTION FINAL EVALUATION TFL RIGHT**

One-way Analysis of Variance (ANOVA)

The P value is 0.4524, considered not significant.

Variation among column means is not significantly greater than expected

by chance.

Bonferroni Multiple Comparisons Test

If the value of t is greater than 2.552 then the P value is less

than 0.05.

Mean

Comparison Difference t P value

================================== ========== ======= ===========

b vs b+pt 68.070 0.8113 ns P>0.05

b vs pt 105.80 1.261 ns P>0.05

b+pt vs pt 37.730 0.4497 ns P>0.05

Mean 95% Confidence Interval

Difference Difference From To

================================== ========== ======= =======

b - b+pt 68.070 -146.08 282.22

b - pt 105.80 -108.35 319.95

b+pt - pt 37.730 -176.42 251.88

Assumption test: Are the standard deviations of the groups equal?

ANOVA assumes that the data are sampled from populations with identical

SDs. This assumption is tested using the method of Bartlett.

Bartlett statistic (corrected) = 1.625

The P value is 0.4437.

Bartlett's test suggests that the differences among the SDs is

not significant.

Assumption test: Are the data sampled from Gaussian distributions?

ANOVA assumes that the data are sampled from populations that follow

Gaussian distributions. This assumption is tested using the method

Kolmogorov and Smirnov:

Group KS P Value Passed normality test?

=============== ====== ======== =======================

b 0.2825 0.0230 No

b+pt 0.2093 >0.10 Yes

pt 0.1851 >0.10 Yes

At least one column failed the normality test with P<0.05.

Consider using a nonparametric test or transforming the data

(i.e. converting to logarithms or reciprocals).

Intermediate calculations. ANOVA table

Source of Degrees of Sum of Mean

variation freedom squares square

============================ ========== ======== ========

Treatments (between columns) 2 57502 28751

Residuals (within columns) 27 950286 35196

---------------------------- ---------- --------

Total 29 1007789

F = 0.8169 =(MStreatment/MSresidual)

Summary of Data

Number Standard

of Standard Error of

Group Points Mean Deviation Mean Median

=============== ====== ======== ========= ======== ========

b 10 533.89 218.74 69.170 496.85

b+pt 10 465.82 194.40 61.475 486.90

pt 10 428.09 141.24 44.665 433.75

95% Confidence Interval

Group Minimum Maximum From To

=============== ======== ======== ========== ==========

b 268.50 934.80 377.43 690.35

b+pt 190.70 673.80 326.76 604.88

pt 236.50 632.40 327.06 529.12

**TFL left BASELINE**

Kruskal-Wallis Test (Nonparametric ANOVA)

The P value is 0.3062, considered not significant.

Variation among column medians is not significantly greater than expected by chance.

The P value is approximate (from chi-square distribution) because

at least one column has two or more identical values.

Calculation detail

Number Sum Mean

of of of

Group Points Ranks Ranks

=============== ======= ======= =======

b 10 188.00 18.800

b+pt 10 148.50 14.850

pt 10 128.50 12.850

Kruskal-Wallis Statistic KW = 2.367 (corrected for ties)

Post tests were not calculated because the P value was greater

than 0.05.

Summary of Data

Number

of

Group Points Median Minimum Maximum

=============== ====== ======== ======== ========

b 10 434.10 156.10 631.10

b+pt 10 322.30 115.60 781.80

pt 10 322.10 154.20 589.30

**BOTOX TFL left t1 vs t2**

Wilcoxon matched-pairs signed-ranks test

Does the median of the differences between b1 and b2 differ signficantly from zero?

The two-tailed P value is 0.0840, considered not quite significant.

Calculation details

Sum of all signed ranks (W) = -35.000

Sum of positive ranks (T+) = 10.000

Sum of negative ranks (T-) = -45.000

Number of pairs = 10

Assumption test: Was the pairing effective?

Nonparametric Spearman correlation coefficient (r) = 0.09091

The one-tailed P value is 0.4056, considered not significant.

Effective pairing results in a significant correlation between the

columns. With these data, the pairing (or matching) appears

not to be effective. The unpaired test may be more appropriate.

Summary of Data

Parameter: b1 b2 Difference

Mean: 420.66 564.87 -144.21

# of points: 10 10 10

Std deviation: 147.41 184.34 232.21

Std error: 46.615 58.293 73.432

Minimum: 156.10 259.30 -625.70

Maximum: 631.10 944.00 117.60

Median: 434.10 529.05 -96.950

Lower 95% CI: 315.22 433.01 -310.31

Upper 95% CI: 526.10 696.73 21.894

**B+PT TFL left t1 vs t2**

Wilcoxon matched-pairs signed-ranks test

Does the median of the differences between Column A and Column B differ signficantly from zero?

The two-tailed P value is 0.0059, considered very significant.

Calculation details

Sum of all signed ranks (W) = -51.000

Sum of positive ranks (T+) = 2.000

Sum of negative ranks (T-) = -53.000

Number of pairs = 10

Assumption test: Was the pairing effective?

Nonparametric Spearman correlation coefficient (r) = 0.8415

The one-tailed P value is 0.0019, considered very significant.

Effective pairing results in a significant correlation between the

columns. With these data, the pairing (or matching) appears

to be effective.

Summary of Data

Parameter: t1 t2 Difference

Mean: 381.98 515.65 -133.67

# of points: 10 10 10

Std deviation: 190.19 211.99 124.22

Std error: 60.142 67.036 39.282

Minimum: 115.60 201.90 -417.00

Maximum: 781.80 892.40 11.000

Median: 322.30 555.80 -113.90

Lower 95% CI: 245.94 364.01 -222.52

Upper 95% CI: 518.02 667.29 -44.815

**PT TFL LEFT t1 vs t2**

Wilcoxon matched-pairs signed-ranks test

Does the median of the differences between FT V1 and FT V2 differ signficantly from zero?

The two-tailed P value is 0.3750, considered not significant.

Calculation details

Sum of all signed ranks (W) = -18.000

Sum of positive ranks (T+) = 18.500

Sum of negative ranks (T-) = -36.500

Number of pairs = 10

Assumption test: Was the pairing effective?

Nonparametric Spearman correlation coefficient (r) = 0.2364

The one-tailed P value is 0.2567, considered not significant.

Effective pairing results in a significant correlation between the

columns. With these data, the pairing (or matching) appears

not to be effective. The unpaired test may be more appropriate.

Summary of Data

Parameter: PT T1 PT T2 Difference

Mean: 341.42 416.22 -74.800

# of points: 10 10 10

Std deviation: 131.07 163.00 211.36

Std error: 41.447 51.546 66.837

Minimum: 154.20 198.20 -513.70

Maximum: 589.30 708.80 232.00

Median: 322.10 369.40 -18.200

Lower 95% CI: 247.67 299.62 -225.98

Upper 95% CI: 435.17 532.82 76.385

**FINAL EVALUATION TFL LEFT AMONG GROUPS**

Kruskal-Wallis Test (Nonparametric ANOVA)

The P value is 0.1594, considered not significant.

Variation among column medians is not significantly greater than expected by chance.

The P value is approximate (from chi-square distribution) because

at least one column has two or more identical values.

Calculation detail

Number Sum Mean

of of of

Group Points Ranks Ranks

=============== ======= ======= =======

b 10 186.00 18.600

b+pt 10 166.00 16.600

pt 10 113.00 11.300

Kruskal-Wallis Statistic KW = 3.673 (corrected for ties)

Post tests were not calculated because the P value was greater

than 0.05.

Summary of Data

Number

of

Group Points Median Minimum Maximum

=============== ====== ======== ======== ========

b 10 529.05 259.30 944.00

b+pt 10 555.80 201.90 892.40

pt 10 369.40 198.20 708.80

**BONFERRONI CORRECTION FINAL EVALUATION TFL LEFT**

One-way Analysis of Variance (ANOVA)

The P value is 0.2145, considered not significant.

Variation among column means is not significantly greater than expected

by chance.

Bonferroni Multiple Comparisons Test

If the value of t is greater than 2.552 then the P value is less

than 0.05.

Mean

Comparison Difference t P value

================================== ========== ======= ===========

b vs b+pt 49.220 0.5869 ns P>0.05

b vs pt 148.65 1.773 ns P>0.05

b+pt vs pt 99.430 1.186 ns P>0.05

Mean 95% Confidence Interval

Difference Difference From To

================================== ========== ======= =======

b - b+pt 49.220 -164.83 263.27

b - pt 148.65 -65.400 362.70

b+pt - pt 99.430 -114.62 313.48

Assumption test: Are the standard deviations of the groups equal?

ANOVA assumes that the data are sampled from populations with identical

SDs. This assumption is tested using the method of Bartlett.

Bartlett statistic (corrected) = 0.5928

The P value is 0.7435.

Bartlett's test suggests that the differences among the SDs is

not significant.

Assumption test: Are the data sampled from Gaussian distributions?

ANOVA assumes that the data are sampled from populations that follow

Gaussian distributions. This assumption is tested using the method

Kolmogorov and Smirnov:

Group KS P Value Passed normality test?

=============== ====== ======== =======================

b 0.2846 0.0212 No

b+pt 0.1658 >0.10 Yes

pt 0.2416 >0.10 Yes

At least one column failed the normality test with P<0.05.

Consider using a nonparametric test or transforming the data

(i.e. converting to logarithms or reciprocals).

Intermediate calculations. ANOVA table

Source of Degrees of Sum of Mean

variation freedom squares square

============================ ========== ======== ========

Treatments (between columns) 2 114686 57343

Residuals (within columns) 27 949398 35163

---------------------------- ---------- --------

Total 29 1064084

F = 1.631 =(MStreatment/MSresidual)

Summary of Data

Number Standard

of Standard Error of

Group Points Mean Deviation Mean Median

=============== ====== ======== ========= ======== ========

b 10 564.87 184.34 58.293 529.05

b+pt 10 515.65 211.99 67.036 555.80

pt 10 416.22 163.00 51.546 369.40

95% Confidence Interval

Group Minimum Maximum From To

=============== ======== ======== ========== ==========

b 259.30 944.00 433.01 696.73

b+pt 201.90 892.40 364.01 667.29

pt 198.20 708.80 299.62 532.82

**BASELINE SUBOCCIPITALIS RIGHT**

Kruskal-Wallis Test (Nonparametric ANOVA)

The P value is 0.1552, considered not significant.

Variation among column medians is not significantly greater than expected by chance.

The P value is approximate (from chi-square distribution) because

at least one column has two or more identical values.

Calculation detail

Number Sum Mean

of of of

Group Points Ranks Ranks

=============== ======= ======= =======

b 10 184.00 18.400

b+ 10 169.00 16.900

b+pt 10 112.00 11.200

Kruskal-Wallis Statistic KW = 3.726 (corrected for ties)

Post tests were not calculated because the P value was greater than 0.05.

Summary of Data

Number

of

Group Points Median Minimum Maximum

=============== ====== ======== ======== ========

b 10 196.25 112.30 382.80

b+ 10 180.85 52.970 292.60

b+pt 10 144.00 109.70 250.90

**SUBOCC right BOTOX t1 vs t2**

Wilcoxon matched-pairs signed-ranks test

Does the median of the differences between Column A and Column B differ signficantly from zero?

The two-tailed P value is 0.0039, considered very significant.

Calculation details

Sum of all signed ranks (W) = -53.000

Sum of positive ranks (T+) = 1.000

Sum of negative ranks (T-) = -54.000

Number of pairs = 10

Assumption test: Was the pairing effective?

Nonparametric Spearman correlation coefficient (r) = 0.8909

The one-tailed P value is 0.0006, considered extremely significant.

Effective pairing results in a significant correlation between the

columns. With these data, the pairing (or matching) appears

to be effective.

Summary of Data

Parameter: t1 t2 Difference

Mean: 206.73 257.67 -50.940

# of points: 10 10 10

Std deviation: 79.804 85.527 41.395

Std error: 25.236 27.046 13.090

Minimum: 112.30 128.60 -127.40

Maximum: 382.80 382.20 0.6000

Median: 196.25 268.25 -40.800

Lower 95% CI: 149.65 196.49 -80.550

Upper 95% CI: 263.81 318.85 -21.330

**SUBOCC right B+FT t1 vs t2**

Wilcoxon matched-pairs signed-ranks test

Does the median of the differences between Column A and Column B

differ signficantly from zero?

The two-tailed P value is 0.0488, considered significant.

Calculation details

Sum of all signed ranks (W) = -39.000

Sum of positive ranks (T+) = 8.000

Sum of negative ranks (T-) = -47.000

Number of pairs = 10

Assumption test: Was the pairing effective?

Nonparametric Spearman correlation coefficient (r) = 0.6322

The one-tailed P value is 0.0272, considered significant.

Effective pairing results in a significant correlation between the

columns. With these data, the pairing (or matching) appears

not to be effective. The unpaired test may be more appropriate.

Summary of Data

Parameter: t1 t2 Difference

Mean: 182.88 216.81 -33.933

# of points: 10 10 10

Std deviation: 80.902 75.463 46.078

Std error: 25.584 23.863 14.571

Minimum: 52.970 93.100 -115.60

Maximum: 292.60 339.80 52.800

Median: 180.85 229.05 -28.400

Lower 95% CI: 125.01 162.83 -66.893

Upper 95% CI: 240.75 270.79 -0.9730

**SUBOCC right PT t1 vs t2**

Wilcoxon matched-pairs signed-ranks test

Does the median of the differences between FT V1 and FT V2 differ signficantly from zero?

The two-tailed P value is 0.0039, considered very significant.

Calculation details

Sum of all signed ranks (W) = -45.000

Sum of positive ranks (T+) = 0.000

Sum of negative ranks (T-) = -45.000

Number of pairs = 9

Note: 1 pair was excluded from calculations because both

values were equal.

Assumption test: Was the pairing effective?

Nonparametric Spearman correlation coefficient (r) = 0.4667

The one-tailed P value is 0.0893, considered not quite significant.

Effective pairing results in a significant correlation between the

columns. With these data, the pairing (or matching) appears

not to be effective. The unpaired test may be more appropriate.

Summary of Data

Parameter: PT t1 PT t2 Difference

Mean: 152.49 191.12 -38.630

# of points: 10 10 10

Std deviation: 42.552 46.307 37.549

Std error: 13.456 14.644 11.874

Minimum: 109.70 146.30 -122.20

Maximum: 250.90 265.90 0.000

Median: 144.00 167.85 -30.700

Lower 95% CI: 122.05 158.00 -65.489

Upper 95% CI: 182.93 224.24 -11.771

**SUBOCCIPITALIS RIGHT FINAL EVALUATION among groups**

Kruskal-Wallis Test (Nonparametric ANOVA)

The P value is 0.1552, considered not significant.

Variation among column medians is not significantly greater than expected by chance.

The P value is approximate (from chi-square distribution) because

exact calculations would have taken too long.

Calculation detail

Number Sum Mean

of of of

Group Points Ranks Ranks

=============== ======= ======= =======

b 10 193.00 19.300

b+pt 10 155.00 15.500

pt 10 117.00 11.700

Kruskal-Wallis Statistic KW = 3.726

Post tests were not calculated because the P value was greater

than 0.05.

Summary of Data

Number

of

Group Points Median Minimum Maximum

=============== ====== ======== ======== ========

b 10 268.25 128.60 382.20

b+pt 10 229.05 93.100 339.80

pt 10 167.85 146.30 265.90

**BONFERRONI CORRECTION SUBOCCIPITALIS RIGHT FINAL EVALUATION among groups**

One-way Analysis of Variance (ANOVA)

The P value is 0.1270, considered not significant.

Variation among column means is not significantly greater than expected

by chance.

Bonferroni Multiple Comparisons Test

If the value of t is greater than 2.552 then the P value is less

than 0.05.

Mean

Comparison Difference t P value

================================== ========== ======= ===========

b vs b+pt 40.860 1.286 ns P>0.05

b vs pt 66.550 2.094 ns P>0.05

b+pt vs pt 25.690 0.8083 ns P>0.05

Mean 95% Confidence Interval

Difference Difference From To

================================== ========== ======= =======

b - b+pt 40.860 -40.269 121.99

b - pt 66.550 -14.579 147.68

b+pt - pt 25.690 -55.439 106.82

Assumption test: Are the standard deviations of the groups equal?

ANOVA assumes that the data are sampled from populations with identical

SDs. This assumption is tested using the method of Bartlett.

Bartlett statistic (corrected) = 3.145

The P value is 0.2076.

Bartlett's test suggests that the differences among the SDs is

not significant.

Assumption test: Are the data sampled from Gaussian distributions?

ANOVA assumes that the data are sampled from populations that follow

Gaussian distributions. This assumption is tested using the method

Kolmogorov and Smirnov:

Group KS P Value Passed normality test?

=============== ====== ======== =======================

b 0.1351 >0.10 Yes

b+pt 0.1553 >0.10 Yes

pt 0.2774 0.0281 No

At least one column failed the normality test with P<0.05.

Consider using a nonparametric test or transforming the data

(i.e. converting to logarithms or reciprocals).

Intermediate calculations. ANOVA table

Source of Degrees of Sum of Mean

variation freedom squares square

============================ ========== ======== ========

Treatments (between columns) 2 22528 11264

Residuals (within columns) 27 136385 5051.3

---------------------------- ---------- --------

Total 29 158913

F = 2.230 =(MStreatment/MSresidual)

Summary of Data

Number Standard

of Standard Error of

Group Points Mean Deviation Mean Median

=============== ====== ======== ========= ======== ========

b 10 257.67 85.527 27.046 268.25

b+pt 10 216.81 75.463 23.863 229.05

pt 10 191.12 46.307 14.644 167.85

95% Confidence Interval

Group Minimum Maximum From To

=============== ======== ======== ========== ==========

b 128.60 382.20 196.49 318.85

b+pt 93.100 339.80 162.83 270.79

pt 146.30 265.90 158.00 224.24

**SUBOCCIP left BASELINE**

Kruskal-Wallis Test (Nonparametric ANOVA)

The P value is 0.1268, considered not significant.

Variation among column medians is not significantly greater than expected by chance.

The P value is approximate (from chi-square distribution) because at least one column has two or more identical values.

Calculation detail

Number Sum Mean

of of of

Group Points Ranks Ranks

=============== ======= ======= =======

B 10 195.00 19.500

B+PT 10 115.00 11.500

PT 10 155.00 15.500

Kruskal-Wallis Statistic KW = 4.131 (corrected for ties)

Post tests were not calculated because the P value was greater

than 0.05.

Summary of Data

Number

of

Group Points Median Minimum Maximum

=============== ====== ======== ======== ========

B 10 191.60 96.100 326.00

B+PT 10 130.30 47.000 252.80

PT 10 154.15 113.60 294.00

**SUBOCC left BOTOX t1 vs t2**

Wilcoxon matched-pairs signed-ranks test

Does the median of the differences between B1 and B2 differ signficantly from zero?

The two-tailed P value is 0.4316, considered not significant.

Calculation details

Sum of all signed ranks (W) = -17.000

Sum of positive ranks (T+) = 19.000

Sum of negative ranks (T-) = -36.000

Number of pairs = 10

Assumption test: Was the pairing effective?

Nonparametric Spearman correlation coefficient (r) = 0.4424

The one-tailed P value is 0.1022, considered not significant.

Effective pairing results in a significant correlation between the

columns. With these data, the pairing (or matching) appears

not to be effective. The unpaired test may be more appropriate.

Summary of Data

Parameter: B1 B2 Difference

Mean: 201.27 217.36 -16.090

# of points: 10 10 10

Std deviation: 73.280 76.717 73.918

Std error: 23.173 24.260 23.375

Minimum: 96.100 124.10 -102.40

Maximum: 326.00 351.40 129.90

Median: 191.60 217.20 -31.300

Lower 95% CI: 148.85 162.48 -68.964

Upper 95% CI: 253.69 272.24 36.784

**B +PT SUBOCCIPITALIS left t1 vs t2**

Wilcoxon matched-pairs signed-ranks test

Does the median of the differences between Column A and Column B

differ signficantly from zero?

The two-tailed P value is 0.0098, considered very significant.

Calculation details

Sum of all signed ranks (W) = -49.000

Sum of positive ranks (T+) = 3.000

Sum of negative ranks (T-) = -52.000

Number of pairs = 10

Assumption test: Was the pairing effective?

Nonparametric Spearman correlation coefficient (r) = 0.8545

The one-tailed P value is 0.0014, considered very significant.

Effective pairing results in a significant correlation between the

columns. With these data, the pairing (or matching) appears

to be effective.

Summary of Data

Parameter: t1 t2 Difference

Mean: 135.91 181.93 -46.020

# of points: 10 10 10

Std deviation: 65.023 92.675 38.849

Std error: 20.562 29.306 12.285

Minimum: 47.000 81.800 -98.600

Maximum: 252.80 351.40 15.600

Median: 130.30 156.85 -48.600

Lower 95% CI: 89.399 115.64 -73.809

Upper 95% CI: 182.42 248.22 -18.231

**SUBOCC left PT t1 vs t2**

Wilcoxon matched-pairs signed-ranks test

Does the median of the differences between FT and FT differ signficantly from zero?

The two-tailed P value is 0.1055, considered not significant.

Calculation details

Sum of all signed ranks (W) = -33.000

Sum of positive ranks (T+) = 11.000

Sum of negative ranks (T-) = -44.000

Number of pairs = 10

Assumption test: Was the pairing effective?

Nonparametric Spearman correlation coefficient (r) = 0.5152

The one-tailed P value is 0.0667, considered not quite significant.

Effective pairing results in a significant correlation between the

columns. With these data, the pairing (or matching) appears

not to be effective. The unpaired test may be more appropriate.

Summary of Data

Parameter: PT t1 PT t2 Difference

Mean: 163.66 202.43 -38.770

# of points: 10 10 10

Std deviation: 55.219 57.130 63.692

Std error: 17.462 18.066 20.141

Minimum: 113.60 110.40 -148.20

Maximum: 294.00 302.40 58.200

Median: 154.15 222.10 -37.600

Lower 95% CI: 124.16 161.56 -84.329

Upper 95% CI: 203.16 243.30 6.789

**SUBOCC left FINAL EVALUATION AMONG GROUPS**

Kruskal-Wallis Test (Nonparametric ANOVA)

The P value is 0.5183, considered not significant.

Variation among column medians is not significantly greater than expected by chance.

The P value is approximate (from chi-square distribution) because

at least one column has two or more identical values.

Calculation detail

Number Sum Mean

of of of

Group Points Ranks Ranks

=============== ======= ======= =======

B 10 176.50 17.650

B+PT 10 131.50 13.150

PT 10 157.00 15.700

Kruskal-Wallis Statistic KW = 1.314 (corrected for ties)

Post tests were not calculated because the P value was greater

than 0.05.

Summary of Data

Number

of

Group Points Median Minimum Maximum

=============== ====== ======== ======== ========

B 10 217.20 124.10 351.40

B+PT 10 156.85 81.800 351.40

PT 10 222.10 110.40 302.40

**BONFERRONI CORRECTION SUBOCC left FINAL EVALUATION AMONG GROUPS**

One-way Analysis of Variance (ANOVA)

The P value is 0.5917, considered not significant.

Variation among column means is not significantly greater than expected

by chance.

Bonferroni Multiple Comparisons Test

If the value of t is greater than 2.552 then the P value is less

than 0.05.

Mean

Comparison Difference t P value

================================== ========== ======= ===========

b vs b+pt 35.430 1.030 ns P>0.05

b vs pt 14.930 0.4342 ns P>0.05

b+pt vs pt -20.500 0.5961 ns P>0.05

Mean 95% Confidence Interval

Difference Difference From To

================================== ========== ======= =======

b - b+pt 35.430 -52.344 123.20

b - pt 14.930 -72.844 102.70

b+pt - pt -20.500 -108.27 67.274

Assumption test: Are the standard deviations of the groups equal?

ANOVA assumes that the data are sampled from populations with identical

SDs. This assumption is tested using the method of Bartlett.

Bartlett statistic (corrected) = 1.934

The P value is 0.3803.

Bartlett's test suggests that the differences among the SDs is

not significant.

Assumption test: Are the data sampled from Gaussian distributions?

ANOVA assumes that the data are sampled from populations that follow

Gaussian distributions. This assumption is tested using the method

Kolmogorov and Smirnov:

Group KS P Value Passed normality test?

=============== ====== ======== =======================

b 0.1517 >0.10 Yes

b+pt 0.1636 >0.10 Yes

pt 0.2261 >0.10 Yes

Intermediate calculations. ANOVA table

Source of Degrees of Sum of Mean

variation freedom squares square

============================ ========== ======== ========

Treatments (between columns) 2 6328.1 3164.1

Residuals (within columns) 27 159642 5912.7

---------------------------- ---------- --------

Total 29 165970

F = 0.5351 =(MStreatment/MSresidual)

Summary of Data

Number Standard

of Standard Error of

Group Points Mean Deviation Mean Median

=============== ====== ======== ========= ======== ========

b 10 217.36 76.717 24.260 217.20

b+pt 10 181.93 92.675 29.306 156.85

pt 10 202.43 57.130 18.066 222.10

95% Confidence Interval

Group Minimum Maximum From To

=============== ======== ======== ========== ==========

b 124.10 351.40 162.48 272.24

b+pt 81.800 351.40 115.64 248.22

pt 110.40 302.40 161.56 243.30

**TRAPEZIUS RIGHT BASELINE**

Kruskal-Wallis Test (Nonparametric ANOVA)

The P value is 0.3995, considered not significant.

Variation among column medians is not significantly greater than expected by chance.

The P value is approximate (from chi-square distribution) because

exact calculations would have taken too long.

Calculation detail

Number Sum Mean

of of of

Group Points Ranks Ranks

=============== ======= ======= =======

B1 10 185.00 18.500

B+PT 10 146.00 14.600

PT 10 134.00 13.400

Kruskal-Wallis Statistic KW = 1.835

Post tests were not calculated because the P value was greater

than 0.05.

Summary of Data

Number

of

Group Points Median Minimum Maximum

=============== ====== ======== ======== ========

B1 10 233.00 119.90 305.06

B+PT 10 179.70 40.500 355.70

PT 10 165.25 99.900 416.80

**TRAPEZIUS RIGHT BOTOX t1 vs t2**

Wilcoxon matched-pairs signed-ranks test

Does the median of the differences between B1 and B2 differ signficantly from zero?

The one-tailed P value is 0.1162, considered not significant.

Calculation details

Sum of all signed ranks (W) = -25.000

Sum of positive ranks (T+) = 15.000

Sum of negative ranks (T-) = -40.000

Number of pairs = 10

Assumption test: Was the pairing effective?

Nonparametric Spearman correlation coefficient (r) = 0.8061

The one-tailed P value is 0.0036, considered very significant.

Effective pairing results in a significant correlation between the

columns. With these data, the pairing (or matching) appears

to be effective.

Summary of Data

Parameter: B1 B2 Difference

Mean: 221.80 238.62 -16.824

# of points: 10 10 10

Std deviation: 59.054 73.271 34.711

Std error: 18.675 23.170 10.976

Minimum: 119.90 151.50 -94.200

Maximum: 305.06 380.20 34.000

Median: 233.00 238.45 -17.000

Lower 95% CI: 179.55 186.21 -41.653

Upper 95% CI: 264.04 291.03 8.005

**TRAPEZIUS RIGHT B+PT T1 vs T2**

Wilcoxon matched-pairs signed-ranks test

Does the median of the differences between Column A and Column B

differ signficantly from zero?

The two-tailed P value is 0.2324, considered not significant.

Calculation details

Sum of all signed ranks (W) = -25.000

Sum of positive ranks (T+) = 15.000

Sum of negative ranks (T-) = -40.000

Number of pairs = 10

Assumption test: Was the pairing effective?

Nonparametric Spearman correlation coefficient (r) = 0.4545

The one-tailed P value is 0.0956, considered not quite significant.

Effective pairing results in a significant correlation between the

columns. With these data, the pairing (or matching) appears

not to be effective. The unpaired test may be more appropriate.

Summary of Data

Parameter: t1 t2 Difference

Mean: 191.31 225.38 -34.066

# of points: 10 10 10

Std deviation: 94.240 102.10 113.30

Std error: 29.801 32.288 35.829

Minimum: 40.500 117.56 -235.80

Maximum: 355.70 422.00 171.90

Median: 179.70 191.20 -28.450

Lower 95% CI: 123.90 152.34 -115.11

Upper 95% CI: 258.72 298.41 46.979

**TRAPEZIUS RIGHT PT t1 vs t2**

Wilcoxon matched-pairs signed-ranks test

Does the median of the differences between FT V1 and FT V2 differ signficantly from zero?

The two-tailed P value is 0.0742, considered not quite significant.

Calculation details

Sum of all signed ranks (W) = -31.000

Sum of positive ranks (T+) = 7.000

Sum of negative ranks (T-) = -38.000

Number of pairs = 9

Note: 1 pair was excluded from calculations because both

values were equal.

Assumption test: Was the pairing effective?

Nonparametric Spearman correlation coefficient (r) = 0.8667

The one-tailed P value is 0.0011, considered very significant.

Effective pairing results in a significant correlation between the

columns. With these data, the pairing (or matching) appears

to be effective.

Summary of Data

Parameter: PT T1 PT t2 Difference

Mean: 192.17 238.93 -46.760

# of points: 10 10 10

Std deviation: 93.330 119.47 72.113

Std error: 29.513 37.781 22.804

Minimum: 99.900 90.800 -197.30

Maximum: 416.80 456.00 42.500

Median: 165.25 216.60 -30.400

Lower 95% CI: 125.41 153.47 -98.343

Upper 95% CI: 258.93 324.39 4.823

**FINAL EVALUATION TRAPEZIUS RIGHT AMONG GROUPS**

Kruskal-Wallis Test (Nonparametric ANOVA)

The P value is 0.8272, considered not significant.

Variation among column medians is not significantly greater than expected by chance.

The P value is approximate (from chi-square distribution) because

exact calculations would have taken too long.

Calculation detail

Number Sum Mean

of of of

Group Points Ranks Ranks

=============== ======= ======= =======

B1 10 168.00 16.800

B+PT 10 144.00 14.400

PT 10 153.00 15.300

Kruskal-Wallis Statistic KW = 0.3794

Post tests were not calculated because the P value was greater

than 0.05.

Summary of Data

Number

of

Group Points Median Minimum Maximum

=============== ====== ======== ======== ========

B1 10 238.45 151.50 380.20

B+PT 10 191.20 117.56 422.00

PT 10 216.60 90.800 456.00

**BONFERRONI CORRECTION FINAL EVALUATION TRAPEZIUS RIGHT AMONG GROUPS**

One-way Analysis of Variance (ANOVA)

The P value is 0.9421, considered not significant.

Variation among column means is not significantly greater than expected

by chance.

Bonferroni Multiple Comparisons Test

If the value of t is greater than 2.552 then the P value is less

than 0.05.

Mean

Comparison Difference t P value

================================== ========== ======= ===========

b vs b+pt 13.244 0.2958 ns P>0.05

b vs pt -0.3100 0.006924 ns P>0.05

b+pt vs pt -13.554 0.3027 ns P>0.05

Mean 95% Confidence Interval

Difference Difference From To

================================== ========== ======= =======

b - b+pt 13.244 -101.03 127.52

b - pt -0.3100 -114.59 113.97

b+pt - pt -13.554 -127.83 100.72

Assumption test: Are the standard deviations of the groups equal?

ANOVA assumes that the data are sampled from populations with identical

SDs. This assumption is tested using the method of Bartlett.

Bartlett statistic (corrected) = 1.984

The P value is 0.3709.

Bartlett's test suggests that the differences among the SDs is not significant.

Assumption test: Are the data sampled from Gaussian distributions?

ANOVA assumes that the data are sampled from populations that follow

Gaussian distributions. This assumption is tested using the method

Kolmogorov and Smirnov:

Group KS P Value Passed normality test?

=============== ====== ======== =======================

b 0.2545 0.0656 Yes

b+pt 0.2034 >0.10 Yes

pt 0.2431 0.0965 Yes

Intermediate calculations. ANOVA table

Source of Degrees of Sum of Mean

variation freedom squares square

============================ ========== ======== ========

Treatments (between columns) 2 1197.4 598.68

Residuals (within columns) 27 270610 10023

---------------------------- ---------- --------

Total 29 271807

F = 0.05973 =(MStreatment/MSresidual)

Summary of Data

Number Standard

of Standard Error of

Group Points Mean Deviation Mean Median

=============== ====== ======== ========= ======== ========

b 10 238.62 73.271 23.170 238.45

b+pt 10 225.38 102.10 32.288 191.20

pt 10 238.93 119.47 37.781 216.60

95% Confidence Interval

Group Minimum Maximum From To

=============== ======== ======== ========== ==========

b 151.50 380.20 186.21 291.03

b+pt 117.56 422.00 152.34 298.41

pt 90.800 456.00 153.47 324.39

**TRAPEZIUS LEFT BASELINE**

Kruskal-Wallis Test (Nonparametric ANOVA)

The P value is 0.4200, considered not significant.

Variation among column medians is not significantly greater than expected by chance.

The P value is approximate (from chi-square distribution) because

at least one column has two or more identical values.

Calculation detail

Number Sum Mean

of of of

Group Points Ranks Ranks

=============== ======= ======= =======

B 10 179.00 17.900

B+PT 10 158.50 15.850

PT 10 127.50 12.750

Kruskal-Wallis Statistic KW = 1.735 (corrected for ties)

Post tests were not calculated because the P value was greater

than 0.05.

Summary of Data

Number

of

Group Points Median Minimum Maximum

=============== ====== ======== ======== ========

B 10 244.10 57.600 433.47

B+PT 10 200.85 49.600 371.70

PT 10 172.45 89.500 286.80

**TRAPEZIUS LEFT BOTOX t1 vs t2**

The two-tailed P value is 0.3223, considered not significant.

Calculation details

Sum of all signed ranks (W) = -20.000

Sum of positive ranks (T+) = 17.500

Sum of negative ranks (T-) = -37.500

Number of pairs = 10

Assumption test: Was the pairing effective?

Nonparametric Spearman correlation coefficient (r) = 0.6242

The one-tailed P value is 0.0302, considered significant.

Effective pairing results in a significant correlation between the

columns. With these data, the pairing (or matching) appears

not to be effective. The unpaired test may be more appropriate.

Summary of Data

Parameter: B1 B2 Difference

Mean: 236.38 251.17 -14.790

# of points: 10 10 10

Std deviation: 119.33 133.96 130.66

Std error: 37.734 42.360 41.317

Minimum: 57.600 98.700 -179.10

Maximum: 433.47 487.40 260.37

Median: 244.10 213.65 -28.135

Lower 95% CI: 151.03 155.35 -108.25

Upper 95% CI: 321.73 346.99 78.670

**TRAPEZIUS LEFT BOTOX+PT t1 vs t2**

Wilcoxon matched-pairs signed-ranks test

Does the median of the differences between Column A and Column B

differ signficantly from zero?

The two-tailed P value is 0.0371, considered significant.

Calculation details

Sum of all signed ranks (W) = -41.000

Sum of positive ranks (T+) = 7.000

Sum of negative ranks (T-) = -48.000

Number of pairs = 10

Assumption test: Was the pairing effective?

Nonparametric Spearman correlation coefficient (r) = 0.7333

The one-tailed P value is 0.0101, considered significant.

Effective pairing results in a significant correlation between the

columns. With these data, the pairing (or matching) appears

to be effective.

Summary of Data

Parameter: t1 t2 Difference

Mean: 212.77 284.02 -71.250

# of points: 10 10 10

Std deviation: 108.74 142.02 110.61

Std error: 34.388 44.911 34.978

Minimum: 49.600 116.90 -318.50

Maximum: 371.70 609.90 67.100

Median: 200.85 262.65 -43.650

Lower 95% CI: 134.98 182.43 -150.37

Upper 95% CI: 290.56 385.61 7.871

**TRAPEZIUS LEFT PT t1 vs t2**

Wilcoxon matched-pairs signed-ranks test

Does the median of the differences between FT V1 and FT V2 differ signficantly from zero?

The two-tailed P value is 0.6953, considered not significant.

Calculation details

Sum of all signed ranks (W) = -9.000

Sum of positive ranks (T+) = 23.000

Sum of negative ranks (T-) = -32.000

Number of pairs = 10

Assumption test: Was the pairing effective?

Nonparametric Spearman correlation coefficient (r) = 0.4909

The one-tailed P value is 0.0774, considered not quite significant.

Effective pairing results in a significant correlation between the

columns. With these data, the pairing (or matching) appears

not to be effective. The unpaired test may be more appropriate.

Summary of Data

Parameter: PT T1 PT T2 Difference

Mean: 174.28 203.67 -29.390

# of points: 10 10 10

Std deviation: 56.573 94.173 86.977

Std error: 17.890 29.780 27.505

Minimum: 89.500 116.90 -193.40

Maximum: 286.80 371.70 51.600

Median: 172.45 165.90 5.900

Lower 95% CI: 133.81 136.31 -91.606

Upper 95% CI: 214.75 271.03 32.826

**TRAPEZIUS LEFT FINAL EVALUATION AMONG GROUPS**

Kruskal-Wallis Test (Nonparametric ANOVA)

The P value is 0.4568, considered not significant.

Variation among column medians is not significantly greater than expected by chance.

The P value is approximate (from chi-square distribution) because

at least one column has two or more identical values.

Calculation detail

Number Sum Mean

of of of

Group Points Ranks Ranks

=============== ======= ======= =======

B 10 158.00 15.800

B+PT 10 178.00 17.800

PT 10 129.00 12.900

Kruskal-Wallis Statistic KW = 1.567 (corrected for ties)

Post tests were not calculated because the P value was greater

than 0.05.

Summary of Data

Number

of

Group Points Median Minimum Maximum

=============== ====== ======== ======== ========

B 10 213.65 98.700 487.40

B+PT 10 262.65 116.90 609.90

PT 10 165.90 116.90 371.70

**BONFERRONI CORRECTION TRAPEZIUS LEFT FINAL EVALUATION AMONG GROUPS**

One-way Analysis of Variance (ANOVA)

The P value is 0.3665, considered not significant.

Variation among column means is not significantly greater than expected

by chance.

Bonferroni Multiple Comparisons Test

If the value of t is greater than 2.552 then the P value is less

than 0.05.

Mean

Comparison Difference t P value

================================== ========== ======= ===========

b vs b+pt -32.850 0.5870 ns P>0.05

b vs pt 47.500 0.8487 ns P>0.05

b+pt vs pt 80.350 1.436 ns P>0.05

Mean 95% Confidence Interval

Difference Difference From To

================================== ========== ======= =======

b - b+pt -32.850 -175.70 110.00

b - pt 47.500 -95.350 190.35

b+pt - pt 80.350 -62.500 223.20

Assumption test: Are the standard deviations of the groups equal?

ANOVA assumes that the data are sampled from populations with identical

SDs. This assumption is tested using the method of Bartlett.

Bartlett statistic (corrected) = 1.540

The P value is 0.4631.

Bartlett's test suggests that the differences among the SDs is

not significant.

Assumption test: Are the data sampled from Gaussian distributions?

ANOVA assumes that the data are sampled from populations that follow

Gaussian distributions. This assumption is tested using the method

Kolmogorov and Smirnov:

Group KS P Value Passed normality test?

=============== ====== ======== =======================

b 0.1981 >0.10 Yes

b+pt 0.1869 >0.10 Yes

pt 0.2507 0.0747 Yes

Intermediate calculations. ANOVA table

Source of Degrees of Sum of Mean

variation freedom squares square

============================ ========== ======== ========

Treatments (between columns) 2 32638 16319

Residuals (within columns) 27 422843 15661

---------------------------- ---------- --------

Total 29 455481

F = 1.042 =(MStreatment/MSresidual)

Summary of Data

Number Standard

of Standard Error of

Group Points Mean Deviation Mean Median

=============== ====== ======== ========= ======== ========

b 10 251.17 133.96 42.360 213.65

b+pt 10 284.02 142.02 44.911 262.65

pt 10 203.67 94.173 29.780 165.90

95% Confidence Interval

Group Minimum Maximum From To

=============== ======== ======== ========== ==========

b 98.700 487.40 155.35 346.99

b+pt 116.90 609.90 182.43 385.61

pt 116.90 371.70 136.31 271.03

**LEVATOR SCAPULAE right BASELINE**

Kruskal-Wallis Test (Nonparametric ANOVA)

The P value is 0.5923, considered not significant.

Variation among column medians is not significantly greater than expected by chance.

The P value is approximate (from chi-square distribution) because

at least one column has two or more identical values.

Calculation detail

Number Sum Mean

of of of

Group Points Ranks Ranks

=============== ======= ======= =======

B 10 163.50 16.350

B+PT 10 169.50 16.950

PT 10 132.00 13.200

Kruskal-Wallis Statistic KW = 1.047 (corrected for ties)

Post tests were not calculated because the P value was greater

than 0.05.

Summary of Data

Number

of

Group Points Median Minimum Maximum

=============== ====== ======== ======== ========

B 10 285.45 80.000 497.20

B+PT 10 290.75 56.800 501.70

PT 10 215.55 95.400 405.00

**LEVATOR SCAPULAE right BOTOX t1 vs t2**

Wilcoxon matched-pairs signed-ranks test

Does the median of the differences between B1 and B2 differ signficantly from zero?

The two-tailed P value is 0.1309, considered not significant.

Calculation details

Sum of all signed ranks (W) = -31.000

Sum of positive ranks (T+) = 12.000

Sum of negative ranks (T-) = -43.000

Number of pairs = 10

Assumption test: Was the pairing effective?

Nonparametric Spearman correlation coefficient (r) = 0.7333

The one-tailed P value is 0.0101, considered significant.

Effective pairing results in a significant correlation between the

columns. With these data, the pairing (or matching) appears

to be effective.

Summary of Data

Parameter: B1 B2 Difference

Mean: 274.59 328.04 -53.450

# of points: 10 10 10

Std deviation: 157.22 176.42 123.37

Std error: 49.717 55.788 39.012

Minimum: 80.000 102.60 -229.80

Maximum: 497.20 664.90 211.70

Median: 285.45 317.80 -76.250

Lower 95% CI: 162.13 201.85 -141.70

Upper 95% CI: 387.05 454.23 34.796

**LEVATOR SCAPULAE right BOTOX+PT t1 vs t2**

Wilcoxon matched-pairs signed-ranks test

Does the median of the differences between Column A and Column B

differ signficantly from zero?

The two-tailed P value is 0.0840, considered not quite significant.

Calculation details

Sum of all signed ranks (W) = -35.000

Sum of positive ranks (T+) = 10.000

Sum of negative ranks (T-) = -45.000

Number of pairs = 10

Assumption test: Was the pairing effective?

Nonparametric Spearman correlation coefficient (r) = 0.7212

The one-tailed P value is 0.0117, considered significant.

Effective pairing results in a significant correlation between the

columns. With these data, the pairing (or matching) appears

to be effective.

Summary of Data

Parameter: t1 t2 Difference

Mean: 283.39 318.60 -35.210

# of points: 10 10 10

Std deviation: 146.37 129.34 106.87

Std error: 46.285 40.900 33.794

Minimum: 56.800 153.50 -142.40

Maximum: 501.70 509.50 243.20

Median: 290.75 330.25 -67.100

Lower 95% CI: 178.69 226.08 -111.65

Upper 95% CI: 388.09 411.12 41.233

**LEVATOR SCAPULAE right PT t1 vs t2**

Wilcoxon matched-pairs signed-ranks test

Does the median of the differences between Column A and Column B differ signficantly from zero?

The two-tailed P value is 0.1309, considered not significant.

Calculation details

Sum of all signed ranks (W) = -31.000

Sum of positive ranks (T+) = 12.000

Sum of negative ranks (T-) = -43.000

Number of pairs = 10

Assumption test: Was the pairing effective?

Nonparametric Spearman correlation coefficient (r) = 0.7212

The one-tailed P value is 0.0117, considered significant.

Effective pairing results in a significant correlation between the

columns. With these data, the pairing (or matching) appears

to be effective.

Summary of Data

Parameter: t1 t2 Difference

Mean: 227.83 262.68 -34.850

# of points: 10 10 10

Std deviation: 90.916 116.38 62.684

Std error: 28.750 36.803 19.822

Minimum: 95.400 150.90 -147.70

Maximum: 405.80 501.70 71.200

Median: 215.55 229.65 -25.150

Lower 95% CI: 162.80 179.43 -79.688

Upper 95% CI: 292.86 345.93 9.988

**LEVATOR SCAPULAE right FINAL EVALUATION AMONG GROUPS**

Kruskal-Wallis Test (Nonparametric ANOVA)

The P value is 0.6227, considered not significant.

Variation among column medians is not significantly greater than expected by chance.

The P value is approximate (from chi-square distribution) because

at least one column has two or more identical values.

Calculation detail

Number Sum Mean

of of of

Group Points Ranks Ranks

=============== ======= ======= =======

B 10 164.00 16.400

B+PT 10 168.00 16.800

PT 10 133.00 13.300

Kruskal-Wallis Statistic KW = 0.9475 (corrected for ties)

Post tests were not calculated because the P value was greater

than 0.05.

Summary of Data

Number

of

Group Points Median Minimum Maximum

=============== ====== ======== ======== ========

B 10 317.80 102.60 664.90

B+PT 10 330.25 153.50 509.50

PT 10 229.65 150.90 501.70

**BONFERRONI CORRECTION LEVATOR SCAPULAE right FINAL EVALUATION AMONG GROUPS**

One-way Analysis of Variance (ANOVA)

The P value is 0.5508, considered not significant.

Variation among column means is not significantly greater than expected

by chance.

Bonferroni Multiple Comparisons Test

If the value of t is greater than 2.552 then the P value is less

than 0.05.

Mean

Comparison Difference t P value

================================== ========== ======= ===========

b vs b+pt 9.440 0.1476 ns P>0.05

b vs pt 65.360 1.022 ns P>0.05

b+pt vs pt 55.920 0.8741 ns P>0.05

Mean 95% Confidence Interval

Difference Difference From To

================================== ========== ======= =======

b - b+pt 9.440 -153.86 172.74

b - pt 65.360 -97.938 228.66

b+pt - pt 55.920 -107.38 219.22

Assumption test: Are the standard deviations of the groups equal?

ANOVA assumes that the data are sampled from populations with identical

SDs. This assumption is tested using the method of Bartlett.

Bartlett statistic (corrected) = 1.674

The P value is 0.4331.

Bartlett's test suggests that the differences among the SDs is

not significant.

Assumption test: Are the data sampled from Gaussian distributions?

ANOVA assumes that the data are sampled from populations that follow

Gaussian distributions. This assumption is tested using the method

Kolmogorov and Smirnov:

Group KS P Value Passed normality test?

=============== ====== ======== =======================

b 0.1352 >0.10 Yes

b+pt 0.1283 >0.10 Yes

pt 0.1977 >0.10 Yes

Intermediate calculations. ANOVA table

Source of Degrees of Sum of Mean

variation freedom squares square

============================ ========== ======== ========

Treatments (between columns) 2 24960 12480

Residuals (within columns) 27 552561 20465

---------------------------- ---------- --------

Total 29 577521

F = 0.6098 =(MStreatment/MSresidual)

Summary of Data

Number Standard

of Standard Error of

Group Points Mean Deviation Mean Median

=============== ====== ======== ========= ======== ========

b 10 328.04 176.42 55.788 317.80

b+pt 10 318.60 129.34 40.900 330.25

pt 10 262.68 116.38 36.803 229.65

95% Confidence Interval

Group Minimum Maximum From To

=============== ======== ======== ========== ==========

b 102.60 664.90 201.85 454.23

b+pt 153.50 509.50 226.08 411.12

pt 150.90 501.70 179.43 345.93

**LEVATOR SCAPULAE left BASELINE**

Kruskal-Wallis Test (Nonparametric ANOVA)

The P value is 0.2441, considered not significant.

Variation among column medians is not significantly greater than expected by chance.

The P value is approximate (from chi-square distribution) because

at least one column has two or more identical values.

Calculation detail

Number Sum Mean

of of of

Group Points Ranks Ranks

=============== ======= ======= =======

B1 10 157.00 15.700

B+PT 10 187.00 18.700

PT 10 121.00 12.100

Kruskal-Wallis Statistic KW = 2.821 (corrected for ties)

Post tests were not calculated because the P value was greater than 0.05.

Summary of Data

Number

of

Group Points Median Minimum Maximum

=============== ====== ======== ======== ========

B1 10 218.15 104.90 524.60

B+PT 10 291.67 47.600 415.50

PT 10 196.95 120.20 253.50

**LEVATOR SCAPULAE left BOTOX t1 vs t2**

Wilcoxon matched-pairs signed-ranks test

Does the median of the differences between B1 and B2

differ signficantly from zero? The two-tailed P value is 0.2754, considered not significant.

Calculation details

Sum of all signed ranks (W) = -23.000

Sum of positive ranks (T+) = 16.000

Sum of negative ranks (T-) = -39.000

Number of pairs = 10

Assumption test: Was the pairing effective?

Nonparametric Spearman correlation coefficient (r) = 0.6848

The one-tailed P value is 0.0173, considered significant.

Effective pairing results in a significant correlation between the

columns. With these data, the pairing (or matching) appears

to be effective.

Summary of Data

Parameter: B1 B2 Difference

Mean: 258.33 308.92 -50.590

# of points: 10 10 10

Std deviation: 154.57 160.63 128.89

Std error: 48.881 50.796 40.760

Minimum: 104.90 109.00 -232.60

Maximum: 524.60 648.10 153.50

Median: 218.15 322.10 -30.650

Lower 95% CI: 147.76 194.02 -142.79

Upper 95% CI: 368.90 423.82 41.608

**LEVATOR SCAPULAE left BOTOX+PT t1 vs t2**

Wilcoxon matched-pairs signed-ranks test

Does the median of the differences between Column A and Column B

differ signficantly from zero?

The two-tailed P value is 0.0137, considered significant.

Calculation details

Sum of all signed ranks (W) = -47.000

Sum of positive ranks (T+) = 4.000

Sum of negative ranks (T-) = -51.000

Number of pairs = 10

Assumption test: Was the pairing effective?

Nonparametric Spearman correlation coefficient (r) = 0.7697

The one-tailed P value is 0.0063, considered very significant.

Effective pairing results in a significant correlation between the

columns. With these data, the pairing (or matching) appears

to be effective.

Summary of Data

Parameter: t1 t2 Difference

Mean: 281.00 382.99 -101.99

# of points: 10 10 10

Std deviation: 124.16 169.27 118.29

Std error: 39.264 53.529 37.408

Minimum: 47.600 172.40 -349.60

Maximum: 415.50 648.10 31.300

Median: 291.67 328.10 -54.835

Lower 95% CI: 192.19 261.91 -186.60

Upper 95% CI: 369.82 504.07 -17.370

**LEVATOR SCAPULAE left PT t1 vs t2**

Wilcoxon matched-pairs signed-ranks test

Does the median of the differences between Column A and Column B

differ signficantly from zero?

The two-tailed P value is 0.3750, considered not significant.

Calculation details

Sum of all signed ranks (W) = -19.000

Sum of positive ranks (T+) = 18.000

Sum of negative ranks (T-) = -37.000

Number of pairs = 10

Assumption test: Was the pairing effective?

Nonparametric Spearman correlation coefficient (r) = 0.7333

The one-tailed P value is 0.0101, considered significant.

Effective pairing results in a significant correlation between the

columns. With these data, the pairing (or matching) appears

to be effective.

Summary of Data

Parameter: t1 t2 Difference

Mean: 192.44 242.33 -49.890

# of points: 10 10 10

Std deviation: 44.578 131.78 107.11

Std error: 14.097 41.673 33.872

Minimum: 120.20 111.00 -302.30

Maximum: 253.50 524.40 55.500

Median: 196.95 193.65 -5.150

Lower 95% CI: 160.55 148.06 -126.51

Upper 95% CI: 224.33 336.60 26.729

**LEVATOR SCAPULAE left FINAL EVALUATION AMONG GROUPS**

Kruskal-Wallis Test (Nonparametric ANOVA)

The P value is 0.1496, considered not significant.

Variation among column medians is not significantly greater than expected by chance.

The P value is approximate (from chi-square distribution) because

at least one column has two or more identical values.

Calculation detail

Number Sum Mean

of of of

Group Points Ranks Ranks

=============== ======= ======= =======

B1 10 158.50 15.850

B+PT 10 191.50 19.150

PT 10 115.00 11.500

Kruskal-Wallis Statistic KW = 3.800 (corrected for ties)

Post tests were not calculated because the P value was greater

than 0.05.

Summary of Data

Number

of

Group Points Median Minimum Maximum

=============== ====== ======== ======== ========

B1 10 322.10 109.00 648.10

B+PT 10 328.10 172.40 648.10

PT 10 193.65 111.00 524.40

**BONFERRONI CORRECTION LEVATOR SCAPULAE left FINAL EVALUATION AMONG GROUPS**

One-way Analysis of Variance (ANOVA)

The P value is 0.1460, considered not significant.

Variation among column means is not significantly greater than expected

by chance.

Bonferroni Multiple Comparisons Test

If the value of t is greater than 2.552 then the P value is less

than 0.05.

Mean

Comparison Difference t P value

================================== ========== ======= ===========

b vs b+pt -74.070 1.070 ns P>0.05

b vs pt 66.590 0.9623 ns P>0.05

b+pt vs pt 140.66 2.033 ns P>0.05

Mean 95% Confidence Interval

Difference Difference From To

================================== ========== ======= =======

b - b+pt -74.070 -250.69 102.55

b - pt 66.590 -110.03 243.21

b+pt - pt 140.66 -35.962 317.28

Assumption test: Are the standard deviations of the groups equal?

ANOVA assumes that the data are sampled from populations with identical

SDs. This assumption is tested using the method of Bartlett.

Bartlett statistic (corrected) = 0.5700

The P value is 0.7520.

Bartlett's test suggests that the differences among the SDs is

not significant.

Assumption test: Are the data sampled from Gaussian distributions?

ANOVA assumes that the data are sampled from populations that follow

Gaussian distributions. This assumption is tested using the method

Kolmogorov and Smirnov:

Group KS P Value Passed normality test?

=============== ====== ======== =======================

b 0.1693 >0.10 Yes

b+pt 0.2121 >0.10 Yes

pt 0.2431 0.0965 Yes

Intermediate calculations. ANOVA table

Source of Degrees of Sum of Mean

variation freedom squares square

============================ ========== ======== ========

Treatments (between columns) 2 99019 49510

Residuals (within columns) 27 646409 23941

---------------------------- ---------- --------

Total 29 745428

F = 2.068 =(MStreatment/MSresidual)

Summary of Data

Number Standard

of Standard Error of

Group Points Mean Deviation Mean Median

=============== ====== ======== ========= ======== ========

b 10 308.92 160.63 50.796 322.10

b+pt 10 382.99 169.27 53.529 328.10

pt 10 242.33 131.78 41.673 193.65

95% Confidence Interval

Group Minimum Maximum From To

=============== ======== ======== ========== ==========

b 109.00 648.10 194.02 423.82

b+pt 172.40 648.10 261.91 504.07

pt 111.00 524.40 148.06 336.60

**FREQUENCY BASELINE**

Kruskal-Wallis Test (Nonparametric ANOVA)

The P value is 0.0762, considered not quite significant.

Variation among column medians is not significantly greater than expected by chance.

The P value is approximate (from chi-square distribution) because

at least one column has two or more identical values.

Calculation detail

Number Sum Mean

of of of

Group Points Ranks Ranks

=============== ======= ======= =======

b 10 145.00 14.500

b+pt 10 203.50 20.350

pt 10 116.50 11.650

Kruskal-Wallis Statistic KW = 5.149 (corrected for ties)

Post tests were not calculated because the P value was greater

than 0.05.

Summary of Data

Number

of

Group Points Median Minimum Maximum

=============== ====== ======== ======== ========

b 10 19.500 13.000 30.000

b+pt 10 29.000 16.000 31.000

pt 10 17.500 15.000 30.000

**FREQUENCY BOTOX T1 VS T2**

Wilcoxon matched-pairs signed-ranks test

Does the median of the differences between b1 and b2 differ signficantly from zero?

The two-tailed P value is 0.0078, considered very significant.

Calculation details

Sum of all signed ranks (W) = 42.000

Sum of positive ranks (T+) = 43.500

Sum of negative ranks (T-) = -1.500

Number of pairs = 9

Note: 1 pair was excluded from calculations because both

values were equal.

Assumption test: Was the pairing effective?

Nonparametric Spearman correlation coefficient (r) = 0.5231

The one-tailed P value is 0.0616, considered not quite significant.

Effective pairing results in a significant correlation between the

columns. With these data, the pairing (or matching) appears

not to be effective. The unpaired test may be more appropriate.

Summary of Data

Parameter: b1 b2 Difference

Mean: 21.500 15.500 6.000

# of points: 10 10 10

Std deviation: 5.255 8.475 6.000

Std error: 1.662 2.680 1.897

Minimum: 13.000 7.000 -2.000

Maximum: 30.000 30.000 18.000

Median: 19.500 12.500 4.500

Lower 95% CI: 17.741 9.437 1.708

Upper 95% CI: 25.259 21.563 10.292

**FREQUENCY BOTOX+PT T1 VS T2**

Wilcoxon matched-pairs signed-ranks test

Does the median of the differences between Column A and Column B differ signficantly from zero?

The two-tailed P value is 0.0039, considered very significant.

Calculation details

Sum of all signed ranks (W) = 45.000

Sum of positive ranks (T+) = 45.000

Sum of negative ranks (T-) = 0.000

Number of pairs = 9

Note: 1 pair was excluded from calculations because both

values were equal.

Assumption test: Was the pairing effective?

Nonparametric Spearman correlation coefficient (r) = 0.7654

The one-tailed P value is 0.0063, considered very significant.

Effective pairing results in a significant correlation between the

columns. With these data, the pairing (or matching) appears

to be effective.

Summary of Data

Parameter: t1 t2 Difference

Mean: 25.900 18.700 7.200

# of points: 10 10 10

Std deviation: 6.082 7.454 3.706

Std error: 1.923 2.357 1.172

Minimum: 16.000 5.000 0.000

Maximum: 31.000 28.000 11.000

Median: 29.000 19.500 8.000

Lower 95% CI: 21.550 13.368 4.549

Upper 95% CI: 30.250 24.032 9.851

**FREQUENCY PT T1 VS T2**

Wilcoxon matched-pairs signed-ranks test

Does the median of the differences between Column A and Column B differ signficantly from zero?

The two-tailed P value is 0.0020, considered very significant.

Calculation details

Sum of all signed ranks (W) = 55.000

Sum of positive ranks (T+) = 55.000

Sum of negative ranks (T-) = 0.000

Number of pairs = 10

Assumption test: Was the pairing effective?

Nonparametric Spearman correlation coefficient (r) = 0.6954

The one-tailed P value is 0.0153, considered significant.

Effective pairing results in a significant correlation between the

columns. With these data, the pairing (or matching) appears

to be effective.

Summary of Data

Parameter: t1 t2 Difference

Mean: 20.400 12.600 7.800

# of points: 10 10 10

Std deviation: 5.522 8.449 5.073

Std error: 1.746 2.672 1.604

Minimum: 15.000 3.000 1.000

Maximum: 30.000 28.000 15.000

Median: 17.500 11.500 6.000

Lower 95% CI: 16.450 6.557 4.171

Upper 95% CI: 24.350 18.643 11.429

**FREQUENCY FINAL EVALUATION among groups**

Kruskal-Wallis Test (Nonparametric ANOVA)

The P value is 0.2383, considered not significant.

Variation among column medians is not significantly greater than expected by chance.

The P value is approximate (from chi-square distribution) because

at least one column has two or more identical values.

Calculation detail

Number Sum Mean

of of of

Group Points Ranks Ranks

=============== ======= ======= =======

b 10 153.50 15.350

b+pt 10 189.00 18.900

pt 10 122.50 12.250

Kruskal-Wallis Statistic KW = 2.868 (corrected for ties)

Post tests were not calculated because the P value was greater

than 0.05.

Summary of Data

Number

of

Group Points Median Minimum Maximum

=============== ====== ======== ======== ========

b 10 12.500 7.000 30.000

b+pt 10 19.500 5.000 28.000

pt 10 11.500 3.000 28.000

**BONFERRONI CORRECTION FREQUENCY FINAL EVALUATION among groups**

One-way Analysis of Variance (ANOVA)

The P value is 0.2627, considered not significant.

Variation among column means is not significantly greater than expected

by chance.

Bonferroni Multiple Comparisons Test

If the value of t is greater than 2.552 then the P value is less

than 0.05.

Mean

Comparison Difference t P value

================================== ========== ======= ===========

b vs b+pt -3.200 0.8790 ns P>0.05

b vs pt 2.900 0.7966 ns P>0.05

b+pt vs pt 6.100 1.676 ns P>0.05

Mean 95% Confidence Interval

Difference Difference From To

================================== ========== ======= =======

b - b+pt -3.200 -12.492 6.092

b - pt 2.900 -6.392 12.192

b+pt - pt 6.100 -3.192 15.392

Assumption test: Are the standard deviations of the groups equal?

ANOVA assumes that the data are sampled from populations with identical

SDs. This assumption is tested using the method of Bartlett.

Bartlett statistic (corrected) = 0.1785

The P value is 0.9146.

Bartlett's test suggests that the differences among the SDs is

not significant.

Assumption test: Are the data sampled from Gaussian distributions?

ANOVA assumes that the data are sampled from populations that follow

Gaussian distributions. This assumption is tested using the method

Kolmogorov and Smirnov:

Group KS P Value Passed normality test?

=============== ====== ======== =======================

b 0.2023 >0.10 Yes

b+pt 0.2161 >0.10 Yes

pt 0.1209 >0.10 Yes

Intermediate calculations. ANOVA table

Source of Degrees of Sum of Mean

variation freedom squares square

============================ ========== ======== ========

Treatments (between columns) 2 186.20 93.100

Residuals (within columns) 27 1789.0 66.259

---------------------------- ---------- --------

Total 29 1975.2

F = 1.405 =(MStreatment/MSresidual)

Summary of Data

Number Standard

of Standard Error of

Group Points Mean Deviation Mean Median

=============== ====== ======== ========= ======== ========

b 10 15.500 8.475 2.680 12.500

b+pt 10 18.700 7.454 2.357 19.500

pt 10 12.600 8.449 2.672 11.500

95% Confidence Interval

Group Minimum Maximum From To

=============== ======== ======== ========== ==========

b 7.000 30.000 9.437 21.563

b+pt 5.000 28.000 13.368 24.032

pt 3.000 28.000 6.557 18.643

**DURATION BASELINE**

**Kruskal-Wallis Test (Nonparametric ANOVA)**

The P value is 0.0773, considered not quite significant.

Variation among column medians is not significantly greater than expected by chance.

The P value is approximate (from chi-square distribution) because

at least one column has two or more identical values.

Calculation detail

Number Sum Mean

of of of

Group Points Ranks Ranks

=============== ======= ======= =======

b 10 153.00 15.300

b+pt 10 200.50 20.050

pt 10 111.50 11.150

Kruskal-Wallis Statistic KW = 5.119 (corrected for ties)

Post tests were not calculated because the P value was greater

than 0.05.

Summary of Data

Number

of

Group Points Median Minimum Maximum

=============== ====== ======== ======== ========

b 10 132.00 31.000 527.00

b+pt 10 160.00 35.000 847.00

pt 10 91.500 24.000 295.00

**Duration botox t1 t2**

Wilcoxon matched-pairs signed-ranks test

Does the median of the differences between b1 and b2 differ signficantly from zero?

The two-tailed P value is 0.0098, considered very significant.

Calculation details

Sum of all signed ranks (W) = 48.000

Sum of positive ranks (T+) = 51.500

Sum of negative ranks (T-) = -3.500

Number of pairs = 10

Assumption test: Was the pairing effective?

Nonparametric Spearman correlation coefficient (r) = 0.6606

The one-tailed P value is 0.0219, considered significant.

Effective pairing results in a significant correlation between the

columns. With these data, the pairing (or matching) appears

to be effective.

Summary of Data

Parameter: b1 b2 Difference

Mean: 197.70 92.500 105.20

# of points: 10 10 10

Std deviation: 174.68 94.253 119.20

Std error: 55.239 29.805 37.694

Minimum: 31.000 10.000 -32.000

Maximum: 527.00 279.00 325.00

Median: 132.00 52.000 59.000

Lower 95% CI: 72.750 25.080 19.937

Upper 95% CI: 322.65 159.92 190.46

**DURATION B+PT T1 T2**

Wilcoxon matched-pairs signed-ranks test

Does the median of the differences between t1 and t2 differ signficantly from zero?

The two-tailed P value is 0.0020, considered very significant.

Calculation details

Sum of all signed ranks (W) = 55.000

Sum of positive ranks (T+) = 55.000

Sum of negative ranks (T-) = 0.000

Number of pairs = 10

Assumption test: Was the pairing effective?

Nonparametric Spearman correlation coefficient (r) = 0.8024

The one-tailed P value is 0.0036, considered very significant.

Effective pairing results in a significant correlation between the

columns. With these data, the pairing (or matching) appears

to be effective.

Summary of Data

Parameter: t1 t2 Difference

Mean: 301.80 149.80 152.00

# of points: 10 10 10

Std deviation: 260.67 141.34 193.84

Std error: 82.432 44.694 61.299

Minimum: 35.000 26.000 9.000

Maximum: 847.00 418.00 629.00

Median: 160.00 102.50 91.000

Lower 95% CI: 115.34 48.702 13.342

Upper 95% CI: 488.26 250.90 290.66

**DURATION PT T1 T2**

Wilcoxon matched-pairs signed-ranks test

Does the median of the differences between Column A and Column B

differ signficantly from zero?

The two-tailed P value is 0.0098, considered very significant.

Calculation details

Sum of all signed ranks (W) = 49.000

Sum of positive ranks (T+) = 52.000

Sum of negative ranks (T-) = -3.000

Number of pairs = 10

Assumption test: Was the pairing effective?

Nonparametric Spearman correlation coefficient (r) = 0.8788

The one-tailed P value is 0.0008, considered extremely significant.

Effective pairing results in a significant correlation between the

columns. With these data, the pairing (or matching) appears

to be effective.

Summary of Data

Parameter: t1 t2 Difference

Mean: 108.50 74.100 34.400

# of points: 10 10 10

Std deviation: 77.648 73.185 32.380

Std error: 24.554 23.143 10.240

Minimum: 24.000 6.000 -16.000

Maximum: 295.00 227.00 76.000

Median: 91.500 58.000 32.500

Lower 95% CI: 52.958 21.750 11.238

Upper 95% CI: 164.04 126.45 57.562

**DURATION FINAL EVALUATION AMONG GROUPS**

Kruskal-Wallis Test (Nonparametric ANOVA)

The P value is 0.3926, considered not significant.

Variation among column medians is not significantly greater than expected by chance.

The P value is approximate (from chi-square distribution) because

at least one column has two or more identical values.

Calculation detail

Number Sum Mean

of of of

Group Points Ranks Ranks

=============== ======= ======= =======

b 10 147.00 14.700

b+pt 10 185.00 18.500

pt 10 133.00 13.300

Kruskal-Wallis Statistic KW = 1.870 (corrected for ties)

Post tests were not calculated because the P value was greater than 0.05.

Summary of Data

Number

of

Group Points Median Minimum Maximum

=============== ====== ======== ======== ========

b 10 52.000 10.000 279.00

b+pt 10 102.50 26.000 418.00

pt 10 58.000 6.000 227.00

**BONFERRONI CORRECTION DURATION FINAL EVALUATION AMONG GROUPS**

One-way Analysis of Variance (ANOVA)

The P value is 0.2720, considered not significant.

Variation among column means is not significantly greater than expected

by chance.

Bonferroni Multiple Comparisons Test

If the value of t is greater than 2.552 then the P value is less

than 0.05.

Mean

Comparison Difference t P value

================================== ========== ======= ===========

b vs b+pt -57.300 1.200 ns P>0.05

b vs pt 18.400 0.3853 ns P>0.05

b+pt vs pt 75.700 1.585 ns P>0.05

Mean 95% Confidence Interval

Difference Difference From To

================================== ========== ======= =======

b - b+pt -57.300 -179.21 64.606

b - pt 18.400 -103.51 140.31

b+pt - pt 75.700 -46.206 197.61

Assumption test: Are the standard deviations of the groups equal?

ANOVA assumes that the data are sampled from populations with identical

SDs. This assumption is tested using the method of Bartlett.

Bartlett statistic (corrected) = 3.819

The P value is 0.1482.

Bartlett's test suggests that the differences among the SDs is

not significant.

Assumption test: Are the data sampled from Gaussian distributions?

ANOVA assumes that the data are sampled from populations that follow

Gaussian distributions. This assumption is tested using the method

Kolmogorov and Smirnov:

Group KS P Value Passed normality test?

=============== ====== ======== =======================

b 0.2317 >0.10 Yes

b+pt 0.2257 >0.10 Yes

pt 0.2140 >0.10 Yes

Intermediate calculations. ANOVA table

Source of Degrees of Sum of Mean

variation freedom squares square

============================ ========== ======== ========

Treatments (between columns) 2 31174 15587

Residuals (within columns) 27 307939 11405

---------------------------- ---------- --------

Total 29 339113

F = 1.367 =(MStreatment/MSresidual)

Summary of Data

Number Standard

of Standard Error of

Group Points Mean Deviation Mean Median

=============== ====== ======== ========= ======== ========

b 10 92.500 94.253 29.805 52.000

b+pt 10 149.80 141.34 44.694 102.50

pt 10 74.100 73.185 23.143 58.000

95% Confidence Interval

Group Minimum Maximum From To

=============== ======== ======== ========== ==========

b 10.000 279.00 25.080 159.92

b+pt 26.000 418.00 48.702 250.90

pt 6.000 227.00 21.750 126.45

**INTENSITY BASELINE**

Kruskal-Wallis Test (Nonparametric ANOVA)

The P value is 0.1378, considered not significant.

Variation among column medians is not significantly greater than expected by chance.

The P value is approximate (from chi-square distribution) because at least one column has two or more identical values.

Calculation detail

Number Sum Mean

of of of

Group Points Ranks Ranks

=============== ======= ======= =======

b 10 182.50 18.250

b+pt 10 169.00 16.900

pt 10 113.50 11.350

Kruskal-Wallis Statistic KW = 3.963 (corrected for ties)

Dunn's Multiple Comparisons Test

Mean Rank

Comparison Difference P value

================================== ========== ===========

b vs. b+pt 1.350 ns P>0.05

b vs. pt 6.900 ns P>0.05

b+pt vs. pt 5.550 ns P>0.05

Summary of Data

Number

of

Group Points Median Minimum Maximum

=============== ====== ======== ======== ========

b 10 8.000 6.000 9.000

b+pt 10 8.000 7.000 8.000

pt 10 7.000 5.000 9.000

**INTENSITY FINAL EVALUATION AMONG GROUPS**

Kruskal-Wallis Test (Nonparametric ANOVA)

The P value is 0.9030, considered not significant.

Variation among column medians is not significantly greater than expected

by chance.

The P value is approximate (from chi-square distribution) because

at least one column has two or more identical values.

Calculation detail

Number Sum Mean

of of of

Group Points Ranks Ranks

=============== ======= ======= =======

b 10 150.50 15.050

b+pt 10 149.50 14.950

pt 10 165.00 16.500

Kruskal-Wallis Statistic KW = 0.2040 (corrected for ties)

Dunn's Multiple Comparisons Test

Mean Rank

Comparison Difference P value

================================== ========== ===========

b vs. b+pt 0.1000 ns P>0.05

b vs. pt -1.450 ns P>0.05

b+pt vs. pt -1.550 ns P>0.05

Summary of Data

Number

of

Group Points Median Minimum Maximum

=============== ====== ======== ======== ========

b 10 5.500 3.000 8.000

b+pt 10 6.000 3.000 8.000

pt 10 6.500 3.000 7.000

**BONFERRONI CORRECTION INTENSITY FINAL EVALUATION AMONG GROUPS**

One-way Analysis of Variance (ANOVA)

The P value is 0.9085, considered not significant.

Variation among column means is not significantly greater than expected

by chance.

Bonferroni Multiple Comparisons Test

If the value of t is greater than 2.552 then the P value is less

than 0.05.

Mean

Comparison Difference t P value

================================== ========== ======= ===========

b vs b+pt 0.1000 0.1437 ns P>0.05

b vs pt -0.2000 0.2873 ns P>0.05

b+pt vs pt -0.3000 0.4310 ns P>0.05

Mean 95% Confidence Interval

Difference Difference From To

================================== ========== ======= =======

b - b+pt 0.1000 -1.677 1.877

b - pt -0.2000 -1.977 1.577

b+pt - pt -0.3000 -2.077 1.477

Assumption test: Are the standard deviations of the groups equal?

ANOVA assumes that the data are sampled from populations with identical

SDs. This assumption is tested using the method of Bartlett.

Bartlett statistic (corrected) = 0.3570

The P value is 0.8365.

Bartlett's test suggests that the differences among the SDs is

not significant.

Assumption test: Are the data sampled from Gaussian distributions?

ANOVA assumes that the data are sampled from populations that follow

Gaussian distributions. This assumption is tested using the method

Kolmogorov and Smirnov:

Group KS P Value Passed normality test?

=============== ====== ======== =======================

b 0.1656 >0.10 Yes

b+pt 0.1960 >0.10 Yes

pt 0.2889 0.0177 No

At least one column failed the normality test with P<0.05.

Consider using a nonparametric test or transforming the data

(i.e. converting to logarithms or reciprocals).

Intermediate calculations. ANOVA table

Source of Degrees of Sum of Mean

variation freedom squares square

============================ ========== ======== ========

Treatments (between columns) 2 0.4667 0.2333

Residuals (within columns) 27 65.400 2.422

---------------------------- ---------- --------

Total 29 65.867

F = 0.09633 =(MStreatment/MSresidual)

Summary of Data

Number Standard

of Standard Error of

Group Points Mean Deviation Mean Median

=============== ====== ======== ========= ======== ========

b 10 5.700 1.636 0.5175 5.500

b+pt 10 5.600 1.647 0.5207 6.000

pt 10 5.900 1.370 0.4333 6.500

95% Confidence Interval

Group Minimum Maximum From To

=============== ======== ======== ========== ==========

b 3.000 8.000 4.529 6.871

b+pt 3.000 8.000 4.422 6.778

pt 3.000 7.000 4.920 6.880

**INTENSITY BOTOX t1 vs t2**

Wilcoxon matched-pairs signed-ranks test

Does the median of the differences between t1 and t2 differ signficantly from zero?

The two-tailed P value is 0.0156, considered significant.

Calculation details

Sum of all signed ranks (W) = 28.000

Sum of positive ranks (T+) = 28.000

Sum of negative ranks (T-) = 0.000

Number of pairs = 7

Note: 3 pairs were excluded from calculations because both values were equal.

Assumption test: Was the pairing effective?

Nonparametric Spearman correlation coefficient (r) = 0.1990

The one-tailed P value is 0.2918, considered not significant.

Effective pairing results in a significant correlation between the

columns. With these data, the pairing (or matching) appears

not to be effective. The unpaired test may be more appropriate.

Summary of Data

Parameter: t1 t2 Difference

Mean: 7.700 5.700 2.000

# of points: 10 10 10

Std deviation: 0.8233 1.636 1.563

Std error: 0.2603 0.5175 0.4944

Minimum: 6.000 3.000 0.000

Maximum: 9.000 8.000 4.000

Median: 8.000 5.500 3.000

Lower 95% CI: 7.111 4.529 0.8816

Upper 95% CI: 8.289 6.871 3.118

**INTENSITY BOTOX+PT t1 vs t2**

Wilcoxon matched-pairs signed-ranks test

Does the median of the differences between t1 and t2 differ signficantly from zero?

The two-tailed P value is 0.0078, considered very significant.

Calculation details

Sum of all signed ranks (W) = 36.000

Sum of positive ranks (T+) = 36.000

Sum of negative ranks (T-) = 0.000

Number of pairs = 8

Note: 2 pairs were excluded from calculations because both

values were equal.

Assumption test: Was the pairing effective?

Nonparametric Spearman correlation coefficient (r) = 0.1816

The one-tailed P value is 0.3037, considered not significant.

Effective pairing results in a significant correlation between the

columns. With these data, the pairing (or matching) appears

not to be effective. The unpaired test may be more appropriate.

Summary of Data

Parameter: t1 t2 Difference

Mean: 7.600 5.600 2.000

# of points: 10 10 10

Std deviation: 0.5164 1.647 1.633

Std error: 0.1633 0.5207 0.5164

Minimum: 7.000 3.000 0.000

Maximum: 8.000 8.000 5.000

Median: 8.000 6.000 2.000

Lower 95% CI: 7.231 4.422 0.8319

Upper 95% CI: 7.969 6.778 3.168

**INTENSITY PT t1 vs t2**

Wilcoxon matched-pairs signed-ranks test

Does the median of the differences between t1 and t2 differ signficantly from zero?

The two-tailed P value is 0.1094, considered not significant.

Calculation details

Sum of all signed ranks (W) = 24.000

Sum of positive ranks (T+) = 30.000

Sum of negative ranks (T-) = -6.000

Number of pairs = 8

Note: 2 pairs were excluded from calculations because both

values were equal.

Assumption test: Was the pairing effective?

Nonparametric Spearman correlation coefficient (r) = 0.4288

The one-tailed P value is 0.1091, considered not significant.

Effective pairing results in a significant correlation between the

columns. With these data, the pairing (or matching) appears

not to be effective. The unpaired test may be more appropriate.

Summary of Data

Parameter: t1 t2 Difference

Mean: 6.800 5.900 0.9000

# of points: 10 10 10

Std deviation: 1.317 1.370 1.370

Std error: 0.4163 0.4333 0.4333

Minimum: 5.000 3.000 -2.000

Maximum: 9.000 7.000 3.000

Median: 7.000 6.500 1.000

Lower 95% CI: 5.858 4.920 -0.08020

Upper 95% CI: 7.742 6.880 1.880
